# Recent Advances of Electrocatalyst and Cell Design for Hydrogen Peroxide Production

**DOI:** 10.1007/s40820-023-01044-2

**Published:** 2023-04-07

**Authors:** Xiao Huang, Min Song, Jingjing Zhang, Tao Shen, Guanyu Luo, Deli Wang

**Affiliations:** 1 Key Laboratory of Material Chemistry for Energy Conversion and Storage, Ministry of Education, School of Chemistry and Chemical Engineering, Huazhong University of Science and Technology, Wuhan, 430074 People’s Republic of China; 2 Hubei Key Laboratory of Processing and Application of Catalytic Materials, College of Chemistry and Chemical Engineering, Huanggang Normal University, Huanggang, 438000 People’s Republic of China

**Keywords:** Electrochemical synthesis, H_2_O_2_, Oxygen reduction reaction, Electrocatalyst

## Abstract

Electrochemical synthesis of H_2_O_2_ via a selective two-electron oxygen reduction reaction has emerged as an attractive alternative to the current energy-consuming anthraquinone process. Herein, the progress on electrocatalysts for H_2_O_2_ generation, including noble metal, transition metal-based, and carbon-based materials, is summarized. At first, the design strategies employed to obtain electrocatalysts with high electroactivity and high selectivity are highlighted. Then, the critical roles of the geometry of the electrodes and the type of reactor in striking a balance to boost the H_2_O_2_ selectivity and reaction rate are systematically discussed. After that, a potential strategy to combine the complementary properties of the catalysts and the reactor for optimal selectivity and overall yield is illustrated. Finally, the remaining challenges and promising opportunities for high-efficient H_2_O_2_ electrochemical production are highlighted for future studies.

## Highlights

- Fundamental principles for designing highly efficient two-electron oxygen reduction reaction catalysts are briefly reviewed.
- Strategies to integrate the components into an efficient device for hydrogen peroxide production are discussed.
- The challenges and perspectives for catalyst and cell design are discussed.

## Graphical abstract

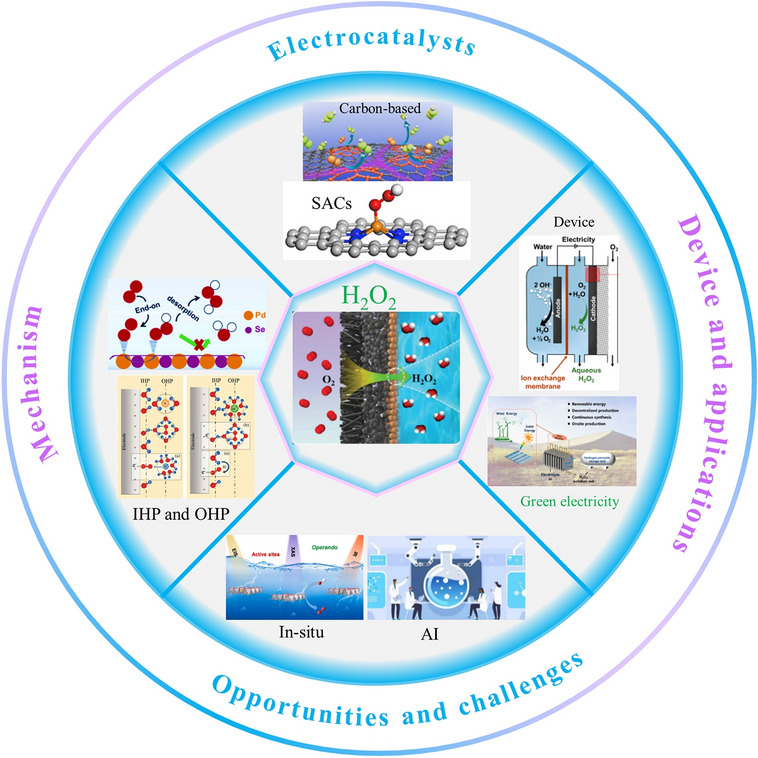

## Introduction

Hydrogen peroxide (H_2_O_2_) is a high-value and environmentally friendly oxidizing agent with a wide range of applications in chemical synthesis, paper and pulp, and wastewater [1, 4]. Currently, 95% of H_2_O_2_ production predominantly depends on the anthraquinone oxidation (AO) process [5−7]. However, this process involves multistep reactions, including a sequential hydrogenation/oxidation of anthraquinone molecules and separations (extraction of H_2_O_2_ from organic solvents), which require enormous energy input and sophisticated facilities [8–10]. The hydrogenation process is initially performed over a Pd catalyst followed by rapid O_2_ oxidation to produce concentrated H_2_O_2_. Then, the H_2_O_2_ is removed by solvent extraction. Despite being able to yield concentrated H_2_O_2_, the large energy input, multiple facilities, and the usage of Pd catalyst increase the cost, and the high concentration of H_2_O_2_ has a risk of storage and transportation, further increasing the cost. These drawbacks have triggered researchers to explore alternative green H_2_O_2_ synthesis technologies. A popular alternative to the traditional AO process is the direct synthesis using H_2_ and O_2_ as the starting reactants [11–15]. This process is performed over noble metal catalysts such as Pd, which can enable continuous and decentralized H_2_O_2_ production. However, the most substantial barrier to the development of this process is the safety issues originating from the explosive nature of H_2_ and O_2_. Thus, to enable large-scale application, other efficient and economic routes for H_2_O_2_ electrosynthesis are highly desirable.

**Fig. 1:**
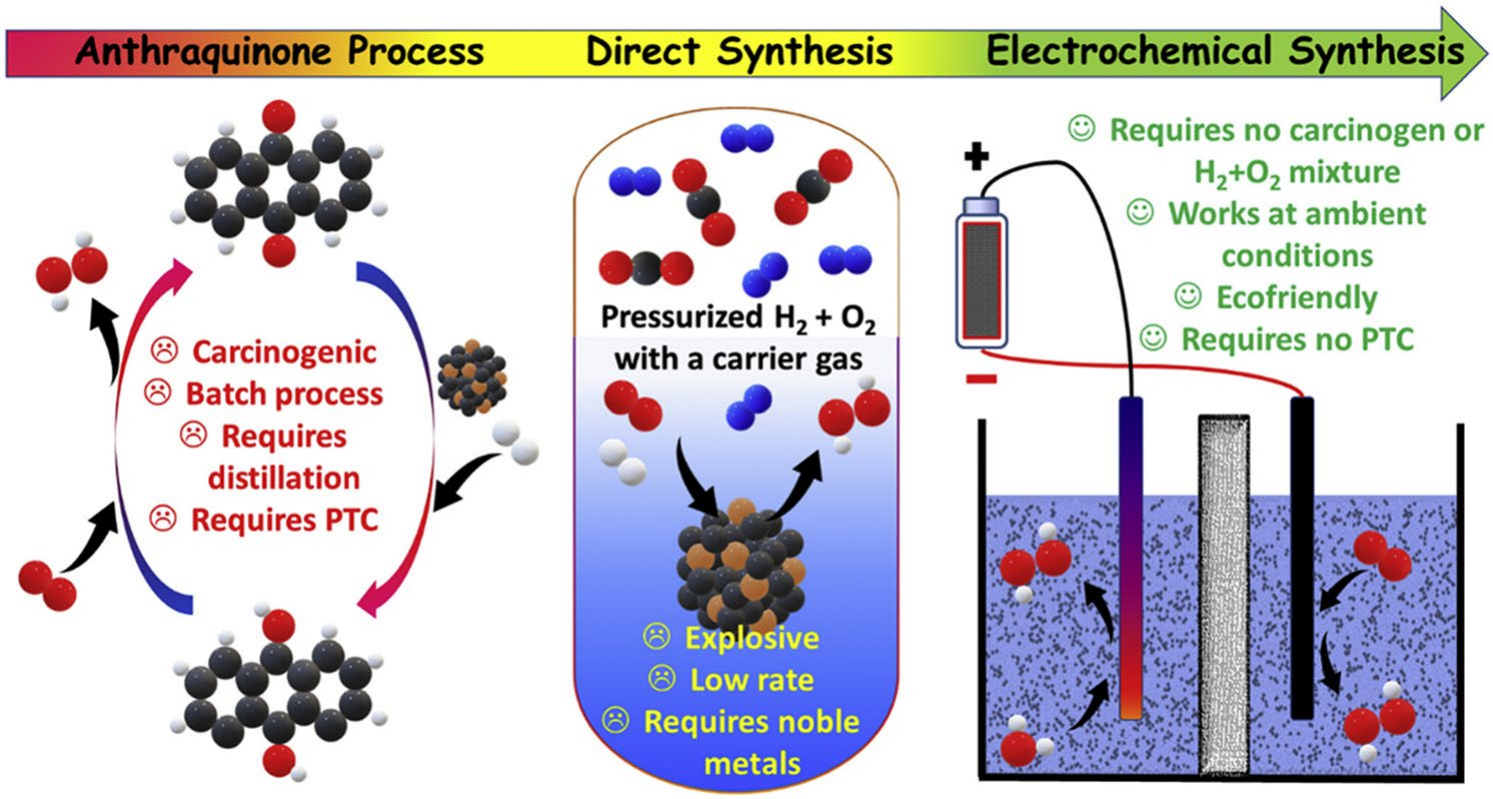
Schematic of H_2_O_2_ production via traditional anthraquinone process, the direct synthesis, and electrochemical synthesis with related demerits and merits. Reprinted with permission [2]. Copyright 2021, Elsevier. (Color figure online)

**Fig. 2:**
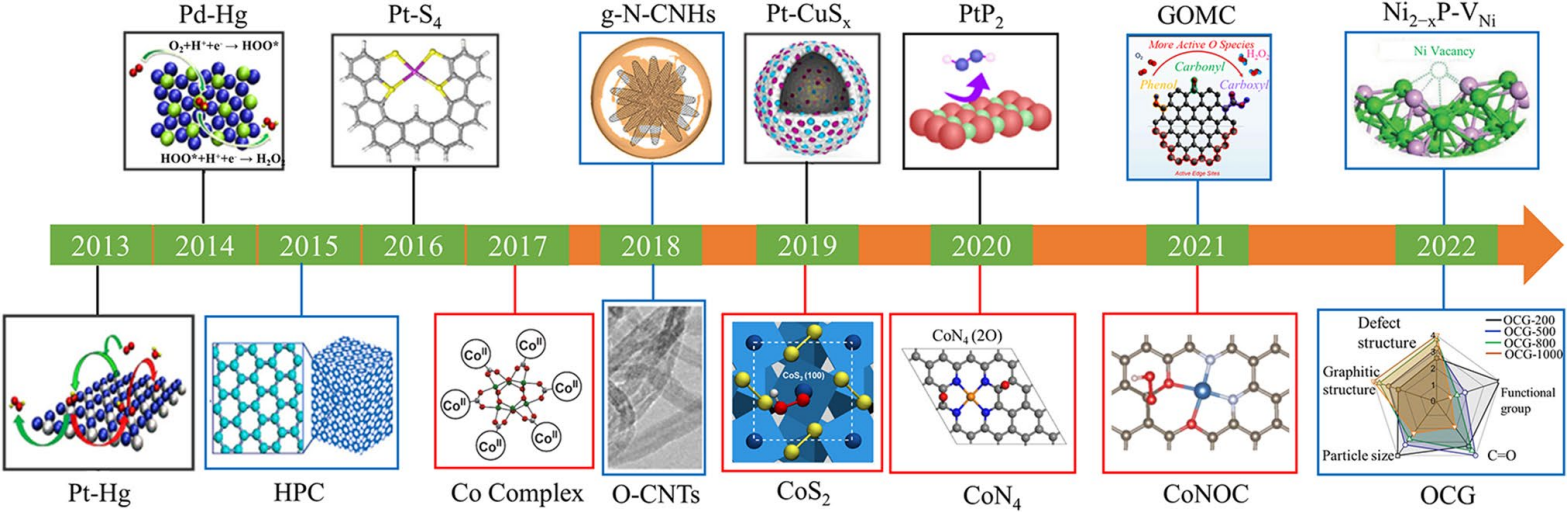
Timeline of some significant findings of 2e^–^ ORR electrocatalyst. PtHg_4_: Reproduced with permission [11]. Copyright 2013, Springer Nature. Pd_2_Hg_5_: Reproduced with permission [61]. Copyright 2014, American Chemical Society. HPC: Reproduced with permission [131]. Copyright 2015, Wiley VCH. Pt-S_4_: Reproduced with permission [79]. Copyright 2016, Springer Nature. Co Complex: Reproduced with permission [93]. Copyright 2017, American Chemical Society. g-N-CNHs and O-CNTs: Reproduced with permission [138, 146]. Copyright 2018, Elsevier Inc. and Copyright 2018, Springer Nature. Pt-CuS_x_ and CoS_2_: Reproduced with permission [77, 98]. Copyright 2019, Elsevier Inc. and Copyright 2019, American Chemical Society. PtP_2_ and CoN_4_: Reproduced with permission [64, 120]. Copyright 2020, Springer Nature. CoNOC and GOMC: Reproduced with permission [121, 132]. Copyright 2021, Springer Nature and Copyright 2021, Elsevier Inc. Ni_2-x_P-V_Ni_ and OCG: Reproduced with permission [100, 134]. Copyright 2022, Wiley VCH. and Copyright 2022, Royal Society of Chemistry. Black: noble metal-based catalysts. Blue: carbon-based catalysts. Red: transition metal-based catalysts. (Color figure online)

Fortunately, the electrochemical strategy through a two-electron oxygen reduction reaction (2e^−^ ORR) pathway provides an attractive route to produce H_2_O_2,_ which is portable and safety [16–19]. Moreover, the electrocatalytic oxygen reduction process just needs water and O_2_ as the starting material, and it could be coupled with renewable energy sources [20–22]. Figure 1 briefly describes the different synthesis methods of H_2_O_2_. The electrochemical strategy is more cost-effective and environmentally friendly than the traditional AO and direct process. In the electrochemical process, the H_2_O_2_ product can be directly generated by the reduction of O_2_ at the cathode. The electrochemical H_2_O_2_ production via a 2e^−^ ORR process was first reported in the 1930s [7, 23]. Since then, the on-site H_2_O_2_ generation from ORR has been widely used for the pulp and paper bleaching process [24–26]. Despite these advantages, the sluggish reaction kinetics and the competing reactions limit the overall energy efficiencies. Thus, the pre-requirements of the H_2_O_2_ electrochemical production process are the rational design of specialized catalysts with high activity, high selectivity, and good stability. The emerging electrocatalyst is divided into noble metal catalysts, transition metal-based catalysts, and carbon-based catalysts. A timeline illustrating the important finding is shown in Fig. 2. H_2_O_2_ is electrochemically produced on the electrode either using a classic H-cell or flow cell with a gas diffusion layer. Therefore, beyond the catalyst-level designs, the electrode and the reaction reactor with the capacity for rapid mass transfer and reactants/products circulating can further enable an efficient H_2_O_2_ production rate.

The research on electrochemical production of H_2_O_2_ via a selective 2e^−^ ORR process is an emerging field. Recent intensive studies have led to the development of various promising catalysts, electrodes, and reaction reactors [20, 27]. Although extensive review articles regarding tailoring the 4e^−^ ORR pathway to the 2e^−^ ORR pathway for H_2_O_2_ production have been reported, the attention paid to the complementary of catalysts with other cell components into an efficient device for H_2_O_2_ production is rare. In this review, the recent advances in the disclosed catalyst design and the improvements made to the electrode and cell design that enables unprecedented H_2_O_2_ electrochemical production are discussed. In the last part, a perspective on some major challenges and opportunities for the rational design of high-efficient catalysts, electrode engineering, and the reaction reactor is presented to accelerate the development of H_2_O_2_ electrochemical production in future studies.

## Fundamentals of H_2_O_2_ Production from Selective Oxygen Reduction Reaction

### Reaction Mechanism of Two-Electron Oxygen Reduction Reaction

**Fig. 3:**
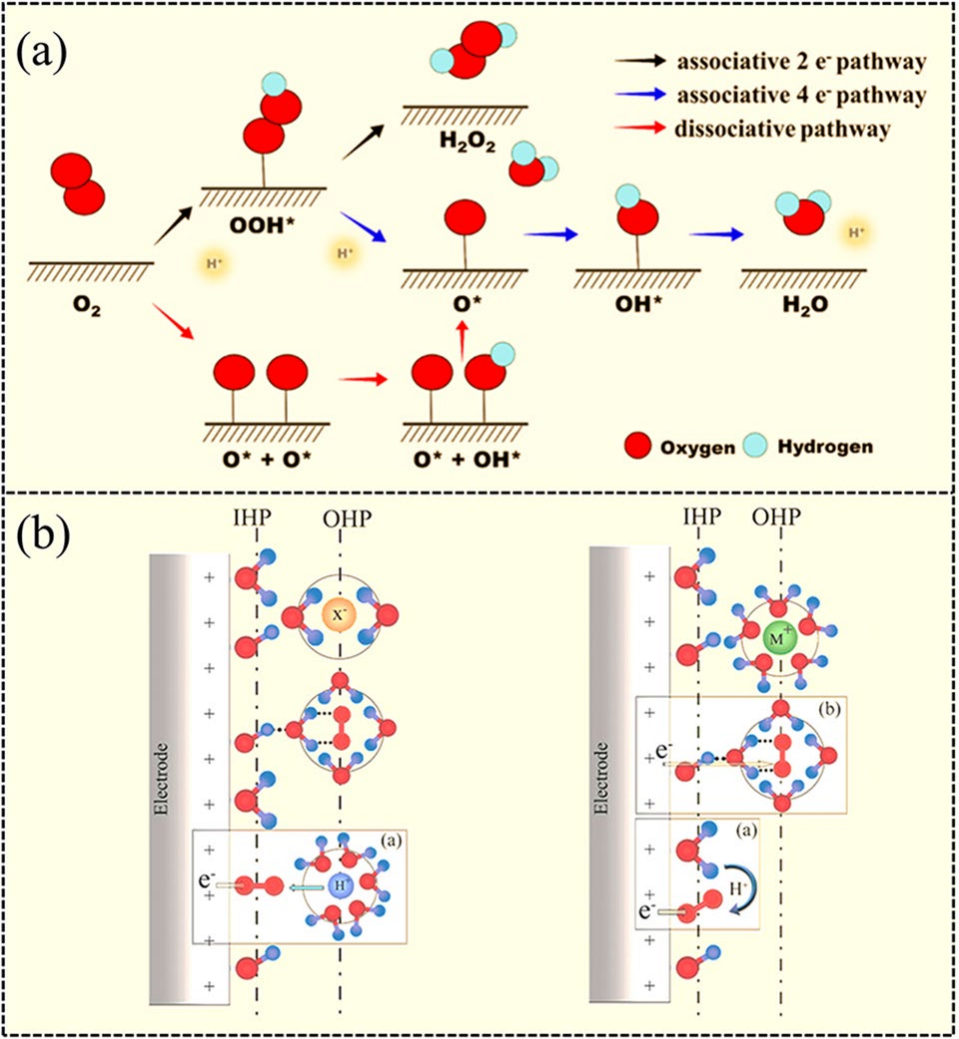
**a** Schematic for the proposed oxygen reduction reaction process. Reproduced with permission [28]. Copyright 2019, American Chemical Society. **b** Schematic illustration of the double-layer structure during ORR in acidic (left) and alkaline (right) conditions. Insets illustrate the inner- and outer-sphere electron transfer processes. Reprinted with permission [29]. Copyright 2011, American Chemical Society. (Color figure online)

In general, the ORR is a multistep process that can proceed with the dissociative mechanism and the associative mechanism [28]. The dissociative mechanism refers to the breakage of O–O bond to form O upon oxygen adsorption, which is reduced successively to OH_ads_ and H_2_O_ads_. The associative mechanism means that the O–O bond is maintained and the final product can be H_2_O or H_2_O_2_ depending on the ability of the catalyst to dissociate the O–O bond in the *OOH intermediate [28–30]. For the ORR to produce H_2_O_2_, it is generally believed that the O–O bond cleavage is unfavorable, and the associative mechanism is dominant. As displayed in Fig. 3a, the O_2_ molecule was firstly adsorbed onto the active sites, followed by a proton-coupled electron transfer process to form *OOH [29, 31]. Subsequently, *OOH would be reduced to the final product of H_2_O_2_, which is desirable for the electrosynthesis of H_2_O_2_ [32]. However, provided that the cleavage of O–O bond occurs, the following reduction of *O would be proceeded, resulting in the formation of undesirable H_2_O [33, 34]. Therefore, protecting the O–O bond and modulating the adsorption energy of *OOH is crucial for achieving high H_2_O_2_ production.

In addition, previous studies have shown that different electrolytes can lead to distinct H_2_O_2_ electrosynthesis performance because solvation effects, surface-adsorbed species (such as OH_ads_ and electrolyte anions), and other factors can affect their ORR pathway. Researchers have studied the ORR performance of Pt/C catalyst in varied pH electrolyte. It was found that in the alkaline medium, the hydroxyl species was strongly adsorbed on the catalyst surface, which is a source of protons and transfers electrons to the water-solvated molecular O_2_ (O_2_·(H_2_O)*n*). The mechanism of the outer-sphere electron transfer for the ORR is dominant in the alkaline medium. Solvated O_2_ and anions filled the outer Helmholtz plane (OHP) (shown in Fig. 3b). O_2_ chemisorption is not a prerequisite, and this process is not specific and can proceed on all catalysts. Therefore, all catalysts can be used for H_2_O_2_ production in alkaline electrolytes. Especially for metal compounds and other catalysts that is semiconductor, this mechanism gives the reasons that can be used for H_2_O_2_ electrosynthesis. The first step of this mechanism is the O_2_ molecules are solvated (O_2_·(H_2_O)*n*), followed by proton and electron transfer to form *OOH intermediates. The selectivity to H_2_O_2_ or H_2_O depends on the cleavage capability of O–O bonds in the *OOH intermediate. For the metal compounds, the binding energy of *OOH on the catalyst surface is weak, thus promoting the desorption of *OOH to form H_2_O_2_. It can be illustrated as:

1 $${\text{M}}-{\text{OH }} + {\text{ }}\left[ {{\text{O}}_{{\text{2}}} \cdot\left( {{\text{H}}_{{\text{2}}} {\text{O}}} \right)n} \right]_{{{\text{aq}}}} + {\text{ e}}^{ - } \to {\text{M}}-{\text{OH }} + {\text{ }}(^{*} {\text{OOH}})_{{{\text{ads}}}} + {\text{ OH}}^{ - } + {\text{ }}\left( {{\text{H}}_{{\text{2}}} {\text{O}}} \right)_{{n - {\text{1}}}}$$

2 $$\left( {^{*} {\text{OOH}}} \right)_{{{\text{ads}}}} + {\text{ e}}^{ - } \to ({\text{HO}}_{{\text{2}}} ^{ - } )_{{{\text{ads}}}}$$

In acidic electrolytes, the high mobility of protons leads to low concentration of adsorbed hydroxyl species on the catalyst surface. O_2_ molecules are chemisorbed on the catalyst surface, then getting electrons from the electrode. This mechanism is called the inner-Helmholtz plane (IHP) process (shown in Fig. 3b). Therefore, the catalysts used for acidic H_2_O_2_ production are conductors, which can facilitate O_2_ chemisorption and electrons transfer. This mechanism elaborates that noble metal-based catalysts and the emerging M–N–C catalysts with high ORR activity can be used for H_2_O_2_ production in acidic electrolyte. The elemental steps for acidic H_2_O_2_ production are:

3 $${\text{O}}_{{2}} \to {\text{O}}_{{2}} ,{\text{ ads}}$$

4 $${\text{O}}_{{{\text{2}},{\text{ ads}}}} + {\text{ e}}^{ - } + {\text{ H}}^{ + } \to \;{}^{*}{\text{OOH}}$$

5 $$^{*} {\text{OOH }} + {\text{ e}}^{ - } + ~{\text{H}}^{ + } \to \;^{*} {\text{OOH}}$$

These ORR pathways give an insight into the pH effect on the H_2_O_2_ electrosynthesis performance and guide us to design catalysts. However, there is rare study about the H_2_O_2_ production mechanism in neutral electrolyte. In the neutral electrolyte, the hydroxyl species is barren as well as the protons. Thus, we should further investigate the ORR pathway to reveal the underlying pH effect on the H_2_O_2_ production.

### Performance Evaluation: Rotating-Ring Disk Electrode Versus Practical Devices

The current H_2_O_2_ electrosynthesis performance evaluation mainly relies on the rotating ring-disk electrode (RRDE) technique in a three-electrode system. This technique is an effective yet facile electrochemical method for quantifying ORR activity, electron transfer number (n), and H_2_O_2_ selectivity (%) in a laboratory setting. Typically, a glassy carbon disk with a Pt ring electrode is used as the working electrode. The ink dripped on the disk was used for H_2_O_2_ production, while the ring is set with 1.2 V (*vs*. RHE) to quantify the H_2_O_2_ amount [35–37]. The negative current at the disk is used to evaluate the electrocatalyst activity. The ORR current on the ring is negligible as the ORR occurs on the disk and the H_2_O_2_ is oxidized on the ring electrode. Therefore, the positive current on the ring is in an index of the H_2_O_2_ selectivity. The electron transfer number (n) and H_2_O_2_ selectivity (%) can be determined by measuring the current at the disk and the ring as the following equations:

6 $$n = \frac{{4\left| {I_{{{\text{disk}}}} } \right|}}{{\left| {I_{{{\text{disk}}}} } \right| + I_{{{\text{ring}}}} /N}}$$

7 $${\text{H}}_{2} {\text{O}}_{2} \% = \frac{{200 \times I_{{{\text{ring}}}} /N}}{{\left| {I_{{{\text{disk}}}} } \right| + I_{{{\text{ring}}}} /N}}$$

where* I*_ring_ and *I*_disk_ refer to ring current and disk current, respectively. *N* represents the collection efficiency of the ring electrode, which is generally obtained via the oxidation–reduction reaction of [Fe(CN)_6_]^4−^/[Fe(CN)_6_]^3−^. In the ORR, the value of n closer to 2 indicates the pathway is toward the 2e^−^ ORR pathway to produce H_2_O_2_, while the value of *n* closer to 4 indicates the 4e^−^ ORR pathway to produce H_2_O. The higher H_2_O_2_ selectivity means that the 2e^−^ ORR pathway is dominant.

The practical H_2_O_2_ production performance of the catalyst can also be evaluated by utilizing electrolyzer configurations. The H_2_O_2_ electrosynthesis performance under such devices can represent the “real world” relative to the RRDE technique and can be used to test catalyst stability and bulk production over an extended time. The H_2_O_2_ concentration can be determined by a traditional Ce(SO_4_)_2_ titration method. The yellow Ce^4+^ ion has a strong absorption peak around 316 nm and can be reduced by H_2_O_2_ to colorless Ce^3+^. Thus, the concentration of H_2_O_2_ will be obtained by the concentration of Ce^4+^ before and after the reaction. Moreover, the faradaic efficiency (FE) in these real devices is calculated to evaluate the catalyst performance (H_2_O_2_ production rate, accumulated H_2_O_2_ concentration, and energy efficiency).

8 $${\text{2Ce}}^{{{4} + }} + {\text{ H}}_{{2}} {\text{O}}_{{2}} \to {\text{2Ce}}^{{{3} + }} + {\text{ 2H}}^{ + } + {\text{ O}}_{{2}}$$

9 $$C_{{{\text{H}}_{2} {\text{O}}_{2} }} { }\left( {{\text{mM}}} \right) = \frac{{V_{{{\text{Ce}}^{4 + } }} \times C_{{{\text{before}}}} {\text{Ce}}^{4 + } - (V_{{{\text{Ce}}^{4 + } }} + V_{{1,{\text{electrolyte}}}} ) \times C_{{{\text{after}}}} {\text{Ce}}^{4 + } }}{{2 \times { }V_{{2,{\text{electrolyte}}}} }}$$

10 $${\text{FE}}\% \, = \frac{2 \times C \times V \times F}{Q}$$

where *C*_H2O2_ is the actual produced H_2_O_2_ concentration, V_Ce4+_ refers to the volume of Ce(SO_4_)_2_, *V*_1, electrolyte_ is the volume of removed electrolyte from the electrolyzer. *C*_before_^Ce4+^ is the initial concentration of Ce(SO_4_)_2_, *C*_after_^Ce4+^ is the final concentration of Ce(SO_4_)_2_ after H_2_O_2_ is added, *V*_2, electrolyte_ refers to the total electrolyte volume. In Eq. 10, *C* is the H_2_O_2_ concentration, *V* is the volume of the electrolyte, F is the faraday constant (96,485 C mol^−1^), and *Q* is the consumed charge. The value of FE is more meaningful to evaluate the H_2_O_2_ electrosynthesis performance compared to the H_2_O_2_% obtained by the RRDE technique.

**Fig. 4:**
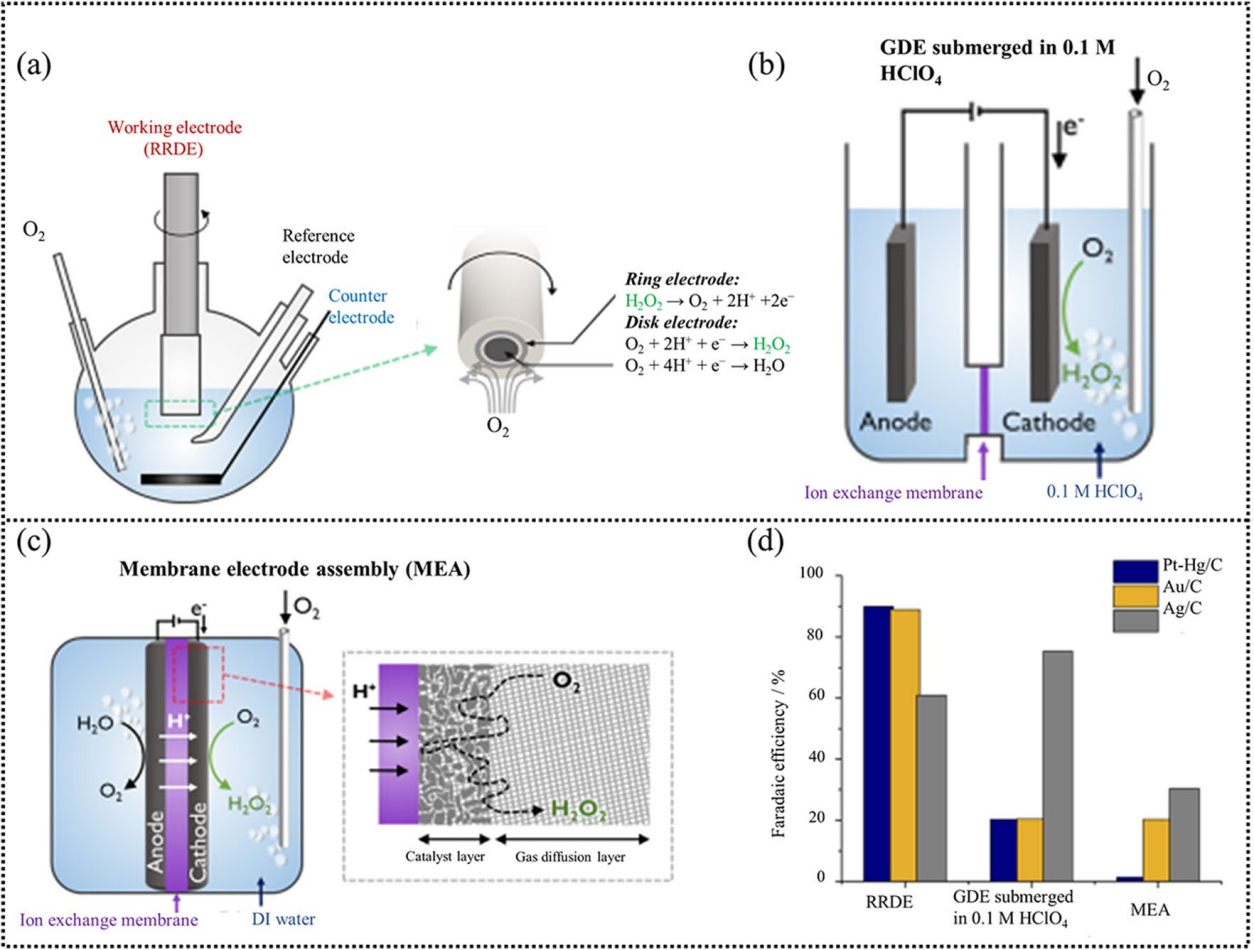
**a** Schematic of a RRDE setup. **b** GDE submerged in 0.1 M HClO_4_. **c** MEA using a three-electrode system. **d** Faradaic efficiency in various laboratory-scale electrochemical cells for H_2_O_2_ electrosynthesis. Reprinted with permission [7]. Copyright 2018, American Chemical Society. (Color figure online)

There could be a distinct gap in the performance evaluation between the RRDE and electrolyzer set-ups because the RRDE technique tends to overperform while the catalyst performance in practical devices shows a more truthful H_2_O_2_ selectivity (faradaic efficiency). A recent work by Yang [7] gave a comparison of the FE of kinds of catalysts on RRDE (Fig. 4a), gas diffusion electrode (submerged, air-breathing) (Fig. 4b), and membrane electrode assembly (MEA) (Fig. 4c). An obvious difference in the selectivity between the RRDE and the practical devices can be clearly seen in Fig. 4d. The main reason behind this phenomenon may be attributed to mass transfer (including oxygen bubbles to the catalyst surface, ions transport, and the H_2_O_2_ transport away from the catalyst surface) and electron transfer (electron transfer from the substrate to the catalyst). For the RRDE technique, the produced H_2_O_2_ at the disk is instantly oxidized at the ring due to the electrode rotation, which can accelerate the H_2_O_2_ transfer and reduce the residence time of H_2_O_2_ on the catalyst surface. Moreover, the rotation can promote ion diffusion and reduce concentration polarization. Nevertheless, the solubility of O_2_ in the electrolyte is very low (70 mg O_2_ L^−1^). The insufficient O_2_ mass transfer restricts the ORR performance. In addition, in the case of the device, the accumulated H_2_O_2_ on the catalyst surface can accelerate the H_2_O_2_ corrosion process and the catalyst can easily deviate from the substrate, resulting in a large deviation in H_2_O_2_ performance evaluation. In this perspective, research efforts under conditions that are more representative of the “real world” are necessary to minimize the gap between fundamental research and practical implementation.

Currently, catalyst development is mainly focused on tuning the 4e^−^ ORR pathway toward the 2e^−^ ORR pathway to produce H_2_O_2_. With the increasing demand for on-site H_2_O_2_ production, the facile electrode preparation has encouraged more search for the integrated electrode with high activity and high selectivity. This review will describe the current insights for H_2_O_2_ electrosynthesis, in which the rational catalyst design, their application, and reactor design strategies are highlighted.

## Catalysts for H_2_O_2_ from the Oxygen Reduction Reaction

The common catalysts used for H_2_O_2_ electrochemical production include noble-metal-based catalysts, transition metal-based catalysts, and carbon-based catalysts. Extensive efforts have been made in the ORR electrocatalysts investigation due to the increasing demand for H_2_O_2_. In this section, the developed catalysts will be concluded and discussed in detail.

### Noble Metal-Based Catalysts

Various noble metal-based catalysts for H_2_O_2_ production have been studied recently including noble metals and their alloys, single-atom catalysts, and so on. Noble metal-based catalysts have been a topic in fuel cells for their high activity and stability [38–41]. It was found that noble metals can produce a trace of H_2_O_2_ when working in fuel cells [42–45]. Therefore, catalysts that undergo incomplete oxygen reduction are less useful but may be a candidate for H_2_O_2_ electrosynthesis [32].

#### Pure Noble Metals

Several noble metals such as Au, Ag, and Hg that have a weak interaction with O_2_ were demonstrated to be able to reduce O_2_ selectively to H_2_O_2_ [46–48]. The produced H_2_O_2_ on the Hg substrate via ORR is stable without further reduction within the operated potential window (*E* ≤ 0.5 V) [49]. In contrast, there was an evident drop in the H_2_O_2_ electrochemical production on the Ag and Au substrate with a more negative potential of 0.025 V [50]. Moreover, a 2e^−^ ORR pathway was observed on the Au cluster, especially on Au(111) and Au(110) surfaces reported in 1983s [51, 52]. Since then, various Au nanostructures were found to be active catalysts for H_2_O_2_ electrochemical production. For example, Au nanoclusters of Au_25_ (consisting of 25 Au atoms) could produce H_2_O_2_ in alkaline media with a high H_2_O_2_ selectivity reaching 90% [53–55]. The enhanced H_2_O_2_ electrosynthesis performance on Au_25_ clusters was strongly related to the Au cluster with low Miller index faces of the crystallographic orientation. Au nanomaterials presented enhanced catalytic activity but low H_2_O_2_ selectivity because of higher surface energy. Therefore, there are few reports about pure noble metals for H_2_O_2_ electrosynthesis due to their harsh synthesis conditions.

#### Noble Metal Alloys

**Fig. 5:**
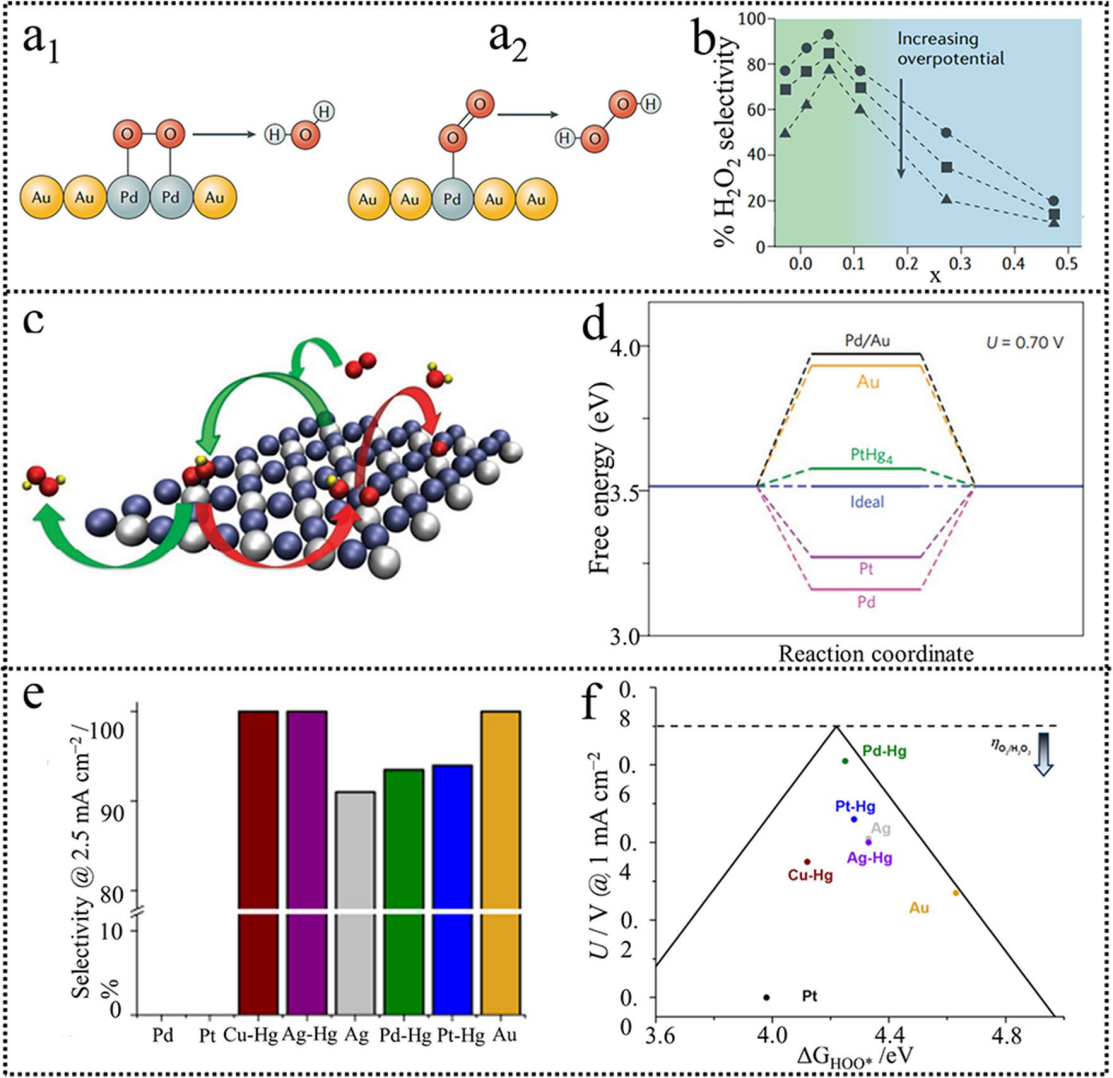
**a** Schematic illustration of Pd_*x*_Au_1−*x*_ showing how discrete single-atom sites change the binding mode from “side-on” to “edge-on”. **b** Selectivity in O_2_ reduction to H_2_O_2_ as a function of Pd content in Pd_x_Au_1−*x*_. Reproduced with permission [1]. Copyright 2019, Springer Nature. **c** Schematic representation of oxygen reduction to H_2_O_2_. **d** Free-energy diagram for oxygen reduction to H_2_O_2_ on a model PtHg_4_ (110) surface. Reproduced with permission [11]. Copyright 2013, Springer Nature. **e** H_2_O_2_ selectivity for different catalysts at 2.5 mA cm^−2^ of total current density. **f** Potential required to reach 1 mA cm^−2^ of kinetic current density to H_2_O_2_ on polycrystalline catalysts as a function of the calculated HOO* binding energy on a model Pd_2_Hg_5_ (001) surface. Reproduced with permission [61]. Copyright 2014, American Chemical Society. (Color figure online)

Pure metals with high ORR activity, such as Pt and Pd, have an extensive application in fuel cells, and they dominate a 4e^−^ ORR pathway. Thus, they can be coupled with weak interacting metals to fabricate bimetallic/ multi-metallic catalysts to improve both electroactivity and H_2_O_2_ selectivity [56–59]. For example, some successful alloys such as Pt-Hg, Pd-Au, and so on, have an extensive study for H_2_O_2_ electrosynthesis. Schiffrin first found that isolating Pd atoms within the Au enhanced the H_2_O_2_ electrosynthesis performance compared to the pure Au and Pd metals [56]. O_2_ is adsorbed “side-on” on the continuous active sites, which makes the cleavage of O−O and H_2_O production easy. Discrete reactive sites could be formed with Au content increasing and O_2_ prefers “end-on” adsorption, which could hinder the O−O breakage and promote the H_2_O_2_ formation (Fig. 5a) [1]. It was demonstrated that when the Pd concentration was increased to 8%, nearly 95% H_2_O_2_ production selectivity was realized (Fig. 5b). Inspired by this, Schiffrin further adopted Au (111) as the substrate and computationally selected other electroactive surfaces containing discrete guest transition metal atoms as Au-M reactive centers. Isolating Pt or Rh atoms by alloying with Au could also enhance H_2_O_2_ production. In addition, Amal et al. [58] reported that Au–Ni core–shell nanoparticles and Au-Pt-Ni core-sandwich-shell nanoparticles also exhibited relatively high ORR activity and H_2_O_2_ selectivity in 0.1 M KOH [60]. Following this, Stephens [11] identified Pt–Hg as a promising candidate (Fig. 5c). It possessed an optimal *OOH binding energy along with a smaller thermodynamic overpotential than the previously reported Pd/Au, indicative of an optimal electrocatalyst for H_2_O_2_ electrosynthesis (Fig. 5d). The PtHg is the only catalyst fulfilling all our criteria for activity, selectivity, and stability among the screening catalysts. Electrochemical results revealed that the as-prepared PtHg_4_ possesses excellent ORR activity with dominant 2e^−^ selectivity under a wide range of potential in 0.1 M HClO_4_. Moreover, PtHg_4_ also remained high activity after 8000 cycles, verifying its stability during the ORR process. Inspired by these results, this group further explored other alloys (CuHg, PdHg, and AgHg) to replace Pt and found that PdHg exhibited more superior ORR activity and H_2_O_2_ selectivity than previously reported PtHg catalyst (Fig. 5e) [61]. DFT calculations revealed that the Pd–Hg exhibited the lowest overpotential (Fig. 5f). The strategy implies that alloying with inert metals can isolate the continuous active sites and facilitate the end-on O_2_ adsorption configuration. Using this strong interaction to prevent the formation of continuous active sites can successfully screen catalysts with high activity and selectivity.

#### Metal-Support Interaction

**Fig. 6:**
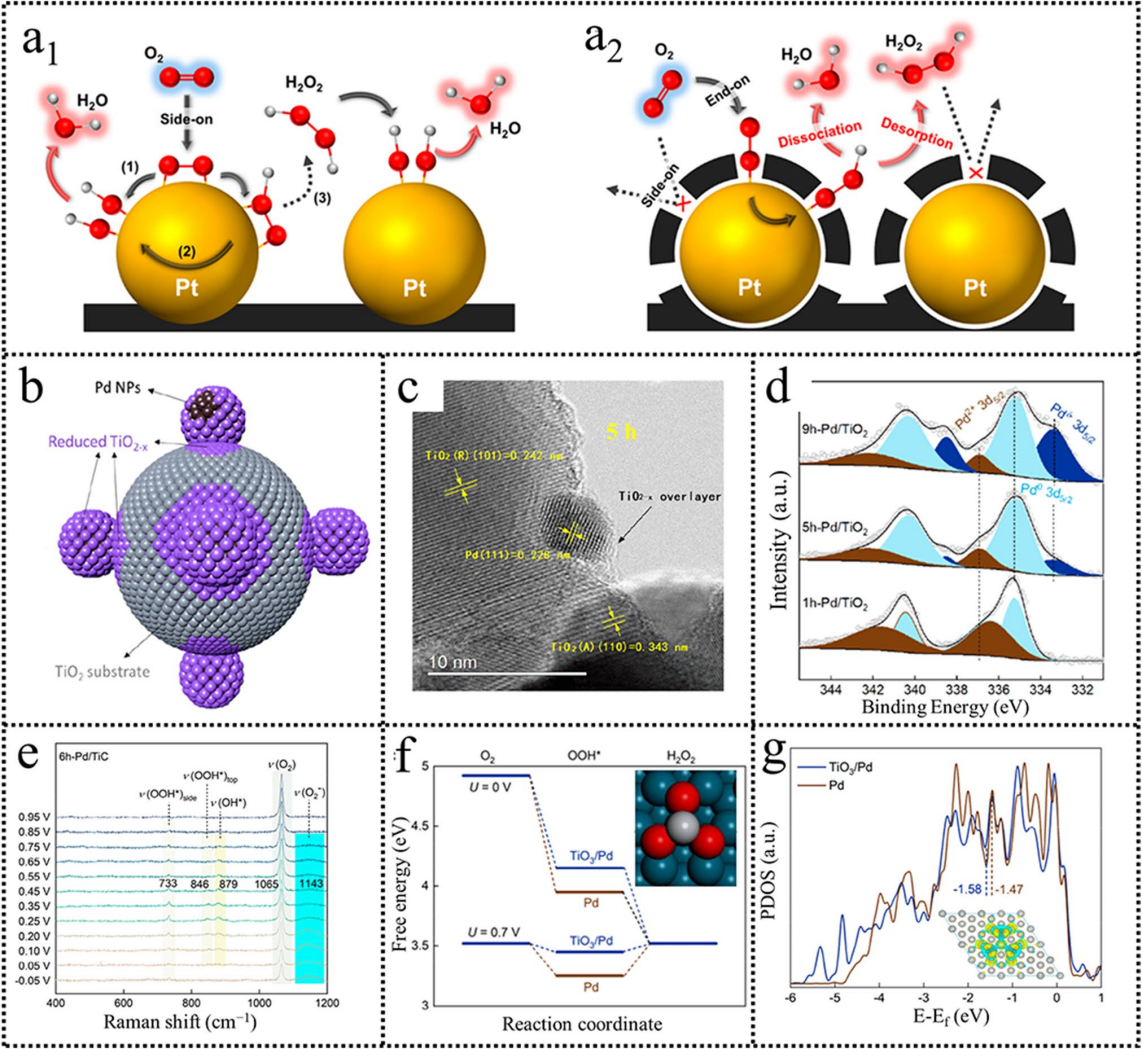
**a** Molecular O_2_ adsorption configuration on Pt/C and C(Pt)/C. Reproduced with permission [62]. Copyright 2014, American Chemical Society. **b** Schematic illustration showing the encapsulation of TiO_2−*x*_ overlayer on Pd NPs. **c** HR-TEM image 5 h–Pd/TiO_2_. **d** XPS spectra of ball-milled Pd/TiO_2-*x*_ samples. **e** In situ Raman spectra recorded over 6 h-Pd/TiC. **f** Free energy diagrams for the two-electron-transfer ORR over TiO_3_/Pd and Pd(111). **g** Projected density of states over TiO_3_/Pd and Pd(111). Reproduced with permission [63]. Copyright 2022, American Chemical Society. (Color figure online)

The transformation from a 4e^−^ ORR pathway to a 2e^−^ ORR pathway for high-performance H_2_O_2_ production is realized by alloying with inert metals. The DFT calculations indicated that the intrinsic property is the O_2_ adsorption model and the *OOH intermediates binding. Therefore, the major challenge for 2e^−^ ORR catalysts design is to switch the O_2_ adsorption mode from “side-on” configuration to “end-on” configuration. Pt/C catalyst has been studied for decades in fuel cells. Unexpectedly, Choi observed that precisely coating amorphous carbon layers on Pt catalysts could induce O_2_ adsorbed with end-on configuration, which remarkably enhanced the H_2_O_2_ electrosynthesis performance (Fig. 6a) [62]. Notably, the optimized C(Pt)C catalyst showed superior catalytic stability originating from the protection of the coated carbon layers compared to the previously studied AuPd alloy. Inspired by this, Liu created an encapsulating Ti oxide overlayer on the Pd species by the ball milling method (Fig. 6b) [63]. A reduced TiO_2-*x*_ layer was found, and the Pd surface was partially oxidized upon increasing the balling time to 5 h (Fig. 6c, d). The electrochemical results indicated when the balling time is 6 h, the obtained catalysts showed excellent H_2_O_2_ electrosynthesis performance. The Pd catalysts encapsulated with Ti oxide overlayer show a much higher mass activity related to the previously reported catalysts and increased long stability. Combined spectroscopy characterization and in situ techniques were used to give an understanding of the reaction mechanism and reaction pathway. In situ Raman spectroscopy results showed that various oxygenated species were observed over the 6 h-Pd/TiC sample (Fig. 6e). The observed signals located at 733 and 846 cm^−1^, which were assigned to the OOH*, corresponded to the bridge site and top site adsorption on the Pd, respectively. The DFT calculations further shed light on the reaction mechanism. The Ti oxide layer reduced the low overpotential (0.07 eV) while increased the negative charge over Pd because the charge was transferred from the Ti oxide layer to Pd active sites (Fig. 6f, g). These results revealed that the electronic and geometric modifications were conducive to optimal adsorption energies of reaction intermediates. The powerful interaction of the support and the noble metals can realize the charge transfer and decrease the surface energy of noble metals.

#### Surface Poisoning

**Fig. 7:**
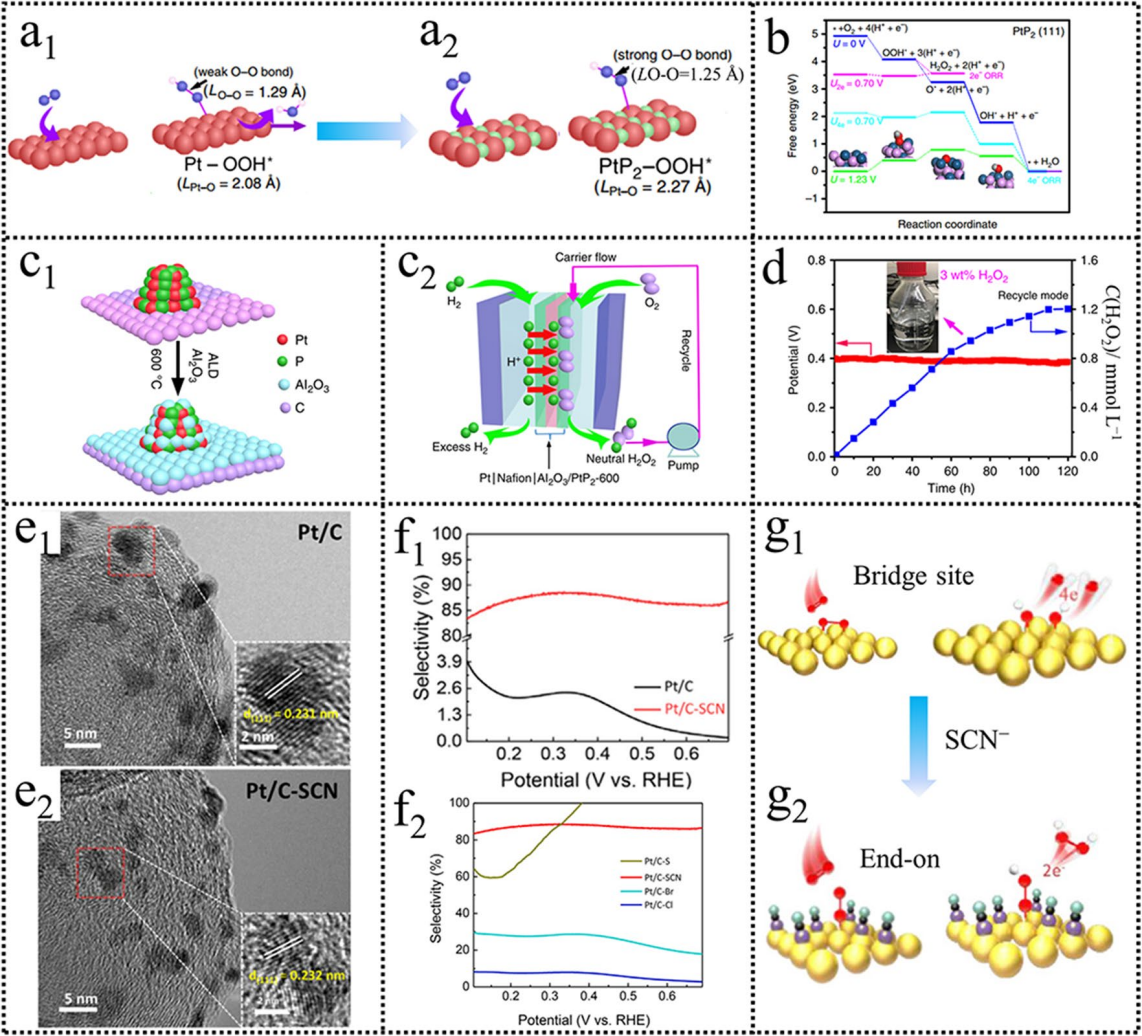
**a** Difference between adsorption behavior of OOH* on bridge site of Pt and PtP_2_. **b** Free-energy diagram for the two-electron and four-electron ORR on PtP_2_. **c** Depiction of Al_2_O_3_ coating by ALD and PEMFC for O_2_-to-H_2_O_2_ production. **d** Time-dependent neutral H_2_O_2_ concentration measured at a constant potential of 0.4 V (*vs*. RHE) for 120 h. Reproduced with permission [64]. Copyright 2020, Springer Nature. **e** TEM images of Pt/C and Pt/C-SCN catalysts. **f, g** H_2_O_2_% selectivity and illustration of ORR transformation pathway for Pt/C catalysts before and after SCN^−^ ion poisoning. Reproduced with permission [68]. Copyright 2021, Elsevier. (Color figure online)

Although the approach of alloying with weak catalysts to isolate the active sites could enable catalysts with superior H_2_O_2_ electrosynthesis performance, a high dosage of metals increases the cost. Alternatively, the incorporation of nonmetal elements, such as P, S, and B, has also been demonstrated as an attractive and effective way for site isolation. Particularly, Li et al. [64] designed and synthesized a PtP_2_ alloy catalyst and explored its electrochemical ORR behavior. PtP_2_ nanocrystals with a uniform size of 3 nm exhibited excellent H_2_O_2_ selectivity compared with Pt nanoparticles (Pt NCs). DFT calculation disclosed the underlying performance improvement. The shifted ORR pathway from 4e^−^ to 2e^−^ originated from the electronegative P incorporation, which changed the electronic density of Pt and increased the separation of adjacent Pt atoms (Fig. 7a).

The DFT results further demonstrated a lower free-energy difference of OOH* to H_2_O_2_ (0.106 eV) than the 0.180 eV for that of *OOH to O*, indicating a suitable *OOH binding energy for H_2_O_2_ formation (Fig. 7b). In addition, to improve electrocatalytic ORR stability, the ultra-small PtP_2_ nanoparticles were treated by atomic layer deposition of an alumina overcoat and post-annealing to suppress PtP_2_ NCs aggregation (Fig. 7c). The coated catalysts achieved a long-term (> 120 h) cycle stability with a high H_2_O_2_ concentration (3.0 wt% with 600 mL) in a neutral medium (pH = 6.6), which can meet the requirement of medical disinfection and sewage disposal (Fig. 7d).

Recently, masking partial active metal surface by adsorbing poisonous ions, such as halogen ions, SCN^−^, CN^−^ and S^2−^, also has been demonstrated as an effective strategy to improve H_2_O_2_ selectivity. In 1999, Markovic et al. [65] studied the effects of Br^−^ ion on Pt (111) surface and found that H_2_O_2_ oxidation currents would increase after Br^−^ ion adsorption. Strongly adsorbed Br^−^ circumvented the formation of paired platinum sites. The effect of S^2−^ ion on the Pt (111) surfaces was further investigated in 0.05 M H_2_SO_4_ and 0.1 M HClO_4_ [66]. The strong adsorption of sulfate ions led to a significant decline in the ORR activity of Pt catalysts and an improvement of 2e^−^ ORR selectivity. Currently, the strategy by the addition of CN^−^ was also proved to improve the H_2_O_2_ selectivity of Pt catalysts [67]. Recently, Niu et al. [68] restudied the influences of SCN^−^, S^2−^, and halide ions on the Pt surface (Fig. 7e). RRDE results revealed that SCN^−^ was the most efficiently poisoning agent for Pt/C catalyst (Fig. 7f). Also, the representative Pt/C-SCN catalyst showed negligible performance loss after long-term stability. A possible mechanism from a 4e^−^ ORR pathway to a 2e^−^ ORR pathway was proposed, as shown in Fig. 7g. Adsorbing halogen ions will poison the noble metal active sites and decrease the continuous active sites, which is almost equivalent to the noble metal alloying catalysts by intrinsically modulating the O_2_ adsorption configuration.

#### Atomically Dispersion

**Fig. 8:**
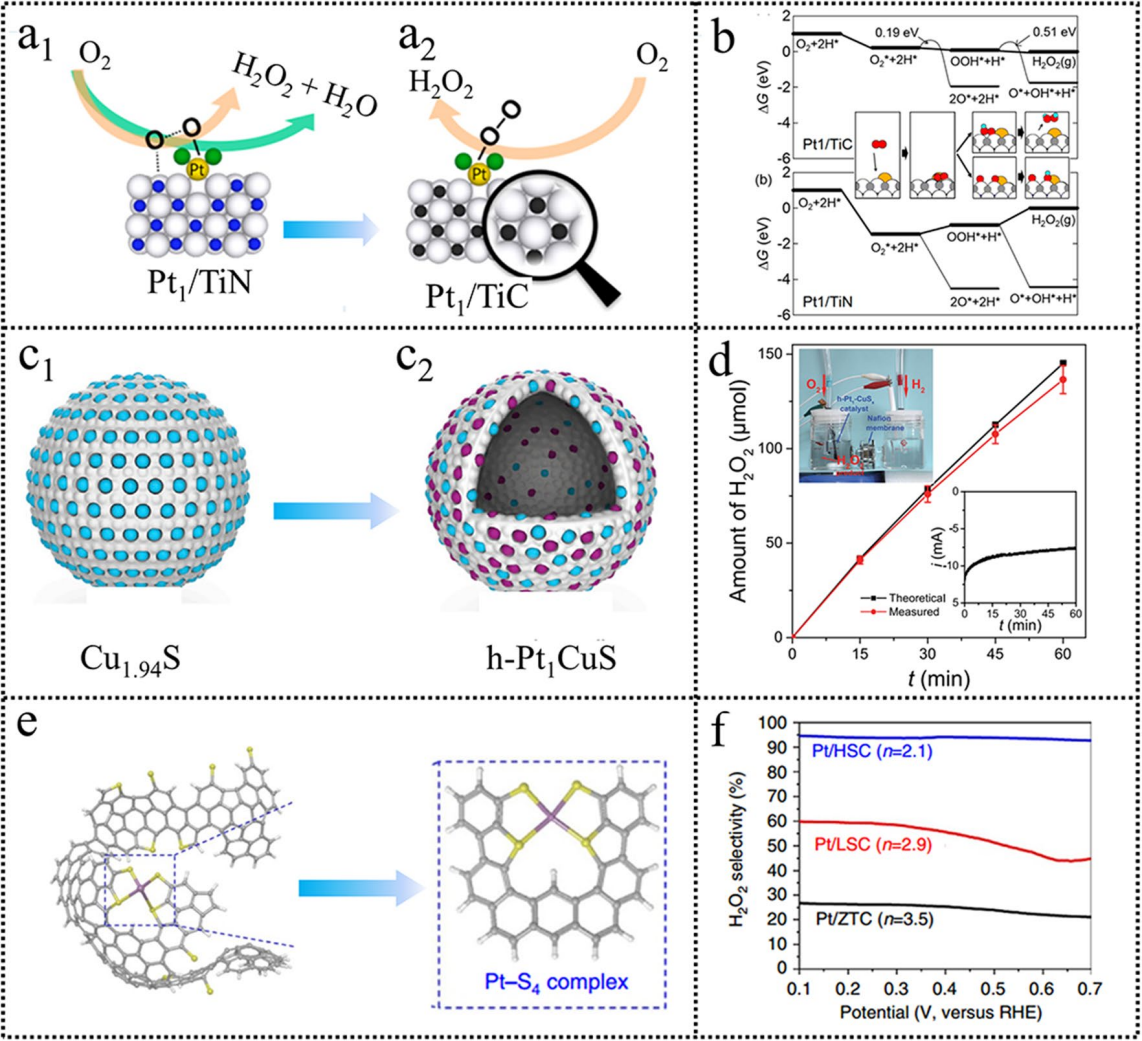
**a**, **b** Schematic of TiN and TiC support effect and free-energy diagrams at 0.2 V for the ORR on Pt/TiC(100) and Pt/TiN(100). Reproduced with permission [76]. Copyright 2016, American Chemical Society. **c**, **d** Schematic illustration of the structure evolution of h-Pt_1_-CuS_*x*_ and H_2_O_2_ generation at a cell. Reproduced with permission [77]. **e**, **f** Proposed atomistic structure of the Pt/HSC based on the buckybowl-like structure and H_2_O_2_ production selectivity. Reproduced with permission [79]. Copyright 2019, Elsevier. (Color figure online)

Alloying with inert atoms or surface poisoning has been demonstrated to be effective to isolate the active site, promoting end-on adsorption for H_2_O_2_ production. Currently, researchers are devoted to noble metal minimization [69–71]. Constructing atomically dispersed catalysts has received extensive attention, which could lower the cost and realize end-on adsorption [72–74]. These factors, such as ligand effect, support effect, and impact of metal loading has an evident influence on the ORR activity and selectivity. Lee et al. [75] studied the effect of Pt loading supported on TiN on the ORR performance. Pt/TiN catalyst with 5 wt% Pt loading exhibited comparable ORR performance to the commercial Pt/C. The ORR current decreased and H_2_O_2_ selectivity increased with the Pt loading decreased. The electrocatalytic activity and H_2_O_2_ selectivity struck a balance when the Pt loading was 0.35 wt%. The ORR current showed a sharp decrease with lower Pt loading although the H_2_O_2_ selectivity reached as high as 90%. Lee et al. further investigated the ORR performance of Pt catalysts supported by different carriers. They found that the H_2_O_2_ selectivity of single Pt atoms anchored on TiN support and TiC support was 53% and 68%, respectively (Fig. 8a) [76]. The higher H_2_O_2_ selectivity on the TiC support was ascribed to the weaker adsorption energy toward *OOH than that on TiN (Fig. 8b). Thus, creating atomically dispersed metal sites on well-defined support is of great importance for H_2_O_2_ selectivity via regulating the O_2_ adsorption configuration.

Although atomically dispersed catalysts exhibit excellent ORR performance to H_2_O_2_ electrosynthesis, they are inclined to aggregation during the preparation process because of their high surface energy. To solve this problem, Li et al. [77] proposed a novel redox-based ion-exchange method (Fig. 8c) and successfully implemented a high concentration of single atomic Pt sites (24.8 at%) on amorphous CuS_*x*_ hollow nanospheres (h-Pt_1_-CuS_*x*_). The Cu_1.94_S support with abundant vacancies has a self-reducibility, which can induce efficient coordination and stabilization with Pt single atoms. Electrochemical results displayed that the h-Pt_1_-CuS_x_ catalyst exhibited a H_2_O_2_ selectivity of 92–96% over a wide potential range of 0.05–0.7 V (*vs*. RHE) in HClO_4_ electrolyte. Importantly, the h-Pt_1_-CuS_x_ catalyst could produce H_2_O_2_ with a yield of 546 mol kg_cat_^−1^ h^−1^ (Fig. 8d), which was the highest value among the reported atomically dispersed electrocatalysts for H_2_O_2_ production.

Metal compounds, such as TiO_2_, TiN, and TiC, can stabilize the noble metal single atoms by coordination interaction or other interactions, but their low electrical conductivity is not conducive to charge transfer during electrocatalysis. Carbon-based materials with high surface area and conductivity in combination with their ligand effect are favorable to stabilizing the noble metal atomically dispersion. Choi et al. [78, 79] reported that a sulfur-doped zeolite-templated carbon could stabilize a relatively high loading of Pt (5 wt%) in the form of isolated atoms (Fig. 8e). Pt species in Pt/HSC (a high S-content) were Pt–S coordination instead of Pt–Pt coordination. The Pt/HSC catalyst exhibited a perfect 2e^−^ ORR pathway (Fig. 8f). After a long cycle stability test, the ORR activity had no significant degradation. Joo et al. [80] recently presented a “trapping-and-immobilizing” strategy toward atomically dispersed noble metal catalysts for H_2_O_2_ synthesis. Precious metal precursors are trapped into a heteroatom-doped carbonaceous layer and “immobilized” by a SiO_2_ layer during thermal activation. This strategy is effective to obtain a series of atomically dispersed precious metal catalysts.

Researchers also study the pH effects on the noble-metal-based catalysts. Lee et al. [41] reported the distinct H_2_O_2_ production performance of Pd–Se–B in various electrolytes. Pd–Se–B showed a surprising activity in neutral electrolyte. Moreover, it exhibited the highest H_2_O_2_ selectivity of 90% in neutral electrolyte than that in 0.5 M H_2_SO_4_ (55%) and 0.1 M KOH (55%) (Table 1). The markable changes of cyclic voltammetry (CV) curves in neutral, acidic, and alkaline electrolytes were indicative of the variable H_2_O_2_ selectivity. Under repeated hydrogen underpotential deposition, Pd^2+^ was reduced to metallic Pd, leading to the increasing ORR activity and decreasing H_2_O_2_ selectivity. While the Pd–Se–B exhibited negligible changes under the potential cycling in neutral electrolyte. This result is against the traditional mechanisms, indicating that the pH effect is of great significance to the H_2_O_2_ electrosynthesis.

**Table 1:** Electrode materials for H_2_O_2_ electrosynthesis at different pH values via different types of electrolytic cells

	Catalyst	Electrolyte	H_2_O_2_ (%)	*n*	Onset potential (V)	Method	Productivity	Method	FE (%)	Refs.
Noble-metal-based catalyst	PtHg_4_	0.1 M HClO_4_	90	2.2	0.6	RRDE				[11]
	h-Pt_1_-CuS_*x*_	0.1 M HClO_4_	95	2.2	0.75	RRDE	546 mmol g_cat_^−1^ h^−1^	H-Cell		[77]
	TiO_2−*x*_/Pd/TiO_2_	0.1 M KOH	90	2.2	0.88	RRDE	594 mg L^−1^ h^−1^	H-cell	85.7	[63]
	Pd–Se–B	0.1 M PBS	90	2.2	0.7	RRDE				[41]
	Pd–Se–B	0.5 M H_2_SO_4_	55	2.7	0.81	RRDE				[41]
	Pd–Se–B	0.1 M KOH	55	2.7	0.9	RRDE				[41]
Transition metal-based catalysts	*o*-CoSe_2_	0.05 M H_2_SO_4_	80	2.4	0.69	RRDE	91 mg L^−1^ h^−1^	H-cell	83	[99]
	Ni_2_Mo_6_S_8_	0.1 M KOH	98	2	0.73	RRDE	1,620 mg L^−1^ h^−1^	H-Cell	85	[104]
	CuCo_0.8_Ni_1.2_S_4_	0.05 M H_2_SO_4_	78	2.5	0.7	RRDE	84 mg L^−1^ h^−1^	H-cell	10	[86]
	a-NiO	0.1 M KOH	90.4	2.2	0.78	RRDE	145 mmol g_cat_^−1^ h^−1^	H-cell	95	[85]
	a-NiB_2_	0.1 M KOH	99	2	0.7	RRDE	65 mg L^−1^ h^−1^	Flow cell	93	[87]
	Ni_2−*x*_P–V_Ni_	0.1 M KOH	95	2.1	0.78	RRDE		Flow cell	80.7	[100]
	Ni_2−*x*_P–V_Ni_	0.1 M PBS						Flow cell	71.1	[100]
	Ni_2−*x*_P–V_Ni_	0.5 M H_2_SO_4_						Flow cell	77.1	[100]
	Co–N–C	0.1 M KOH	62	3.1	0.95	RRDE	193 mmol g_cat_^−1^ h^−1^	H-Cell		[118]
	Co_1_–NG(O)	0.1 M KOH	82	2.3	0.8	RRDE	242 mg L^−1^ h^−1^	H-cell		[120]
	Co_1_–NG(O)	0.1 M HClO_4_	50	3.25	0.78	RRDE				[120]
	Co_1_–NG(O)	0.1 M PBS	66	3.3	0.68	RRDE				[120]
	IS-NiOC	0.1 M PBS	80	2.4	0.5	RRDE	59 mg cm^−2^ h^−1^	Flow cell	90.4	[161]
	CoN_4_/VG	0.1 M HClO_4_	98	2.0	0.7	H-cell	92 mg L^−1^ h^−1^			[113]
	CoN_4_/VG	0.1 M HClO_4_	85	2.3	− 1.02	Flow-cell	1,100 mg L^−1^ h^−1^			[113]
Carbon materials	O-CNTs	0.1 M KOH	90	2.2	0.8	RRDE	3,950 mg L^−1^ h^−1^	H-cell	90	[146]
	O-CNTs	0.1 M PBS	85	2.3	0.51	RRDE				[146]
	O-CNTs	0.1 M HClO_4_	53	3.2	0.3	RRDE				[146]
	NCMK3IL	0.5 M H_2_SO_4_	95	2.1	0.44	RRDE	160 mmol g_cat_^−1^ h^−1^	H-cell	72.5	[25]
	B–C	0.1 M KOH	90	2.2	0.773	RRDE				[142]
	B–C	0.1 M Na_2_SO_4_	80	2.4	0.5	RRDE				[142]
	B–C	1 M KOH			0.685			Flow cell	85.1	[142]
	B–C	1 M Na_2_SO_4_			0.277			Flow cell	83.2	[142]
	B–C	Solid-electrolyte			2.55		1100 mg L^−1^ h^−1^	Solid-electrolyte	85.5	[142]

### Transition Metal-Based Catalysts

Compared with the noble metal-based catalysts, transition metal-based catalysts (such as Fe, Co, Ni, and Cu) have aroused interest due to their earth-abundant and tunable electronic structure of the central transition metal atoms [81–84]. Transition metal-based materials, including metal complexes, metal compounds, and transition nitrogen-doped carbon materials, have been reported as efficient electrocatalysts for O_2_ reduction to H_2_O_2_ [85–87].

#### Metal Complexes

Porphyrin iron in hemoglobin can act as an oxygen carrier for transport, which can effectively convert inspired oxygen into H_2_O. Subsequently, a series of metal (Fe, Co, Cu, Ni) porphyrins, phthalocyanines, and their derivatives are studied successively as ORR catalysts [88–92]. Especially, the represented Fe and Co macrocyclic compounds showed good ORR activity [93–96]. Factors that direct the reaction pathway are the types of metal–oxygen intermediates, such as binuclear peroxo and electronic and steric effects of ligands in catalysts [97]. However, the high cost and unsatisfactory durability of these catalysts largely prohibited their practical applications.

#### Metal Compounds

Jin et al. [98] demonstrated that cobalt pyrite (CoS_2_) exhibited a well 2e^−^ ORR performance in acidic solution. The H_2_O_2_ selectivity was chemically quantified to be 70% at 0.5 V (*vs*. RHE). The modest binding of *OOH on the Co site on the (100) plane of CoS_2_ and the kinetically disfavored O−O bond scission promoted H_2_O_2_ electrosynthesis because S could break Co ensemble active sites.

**Fig. 9:**
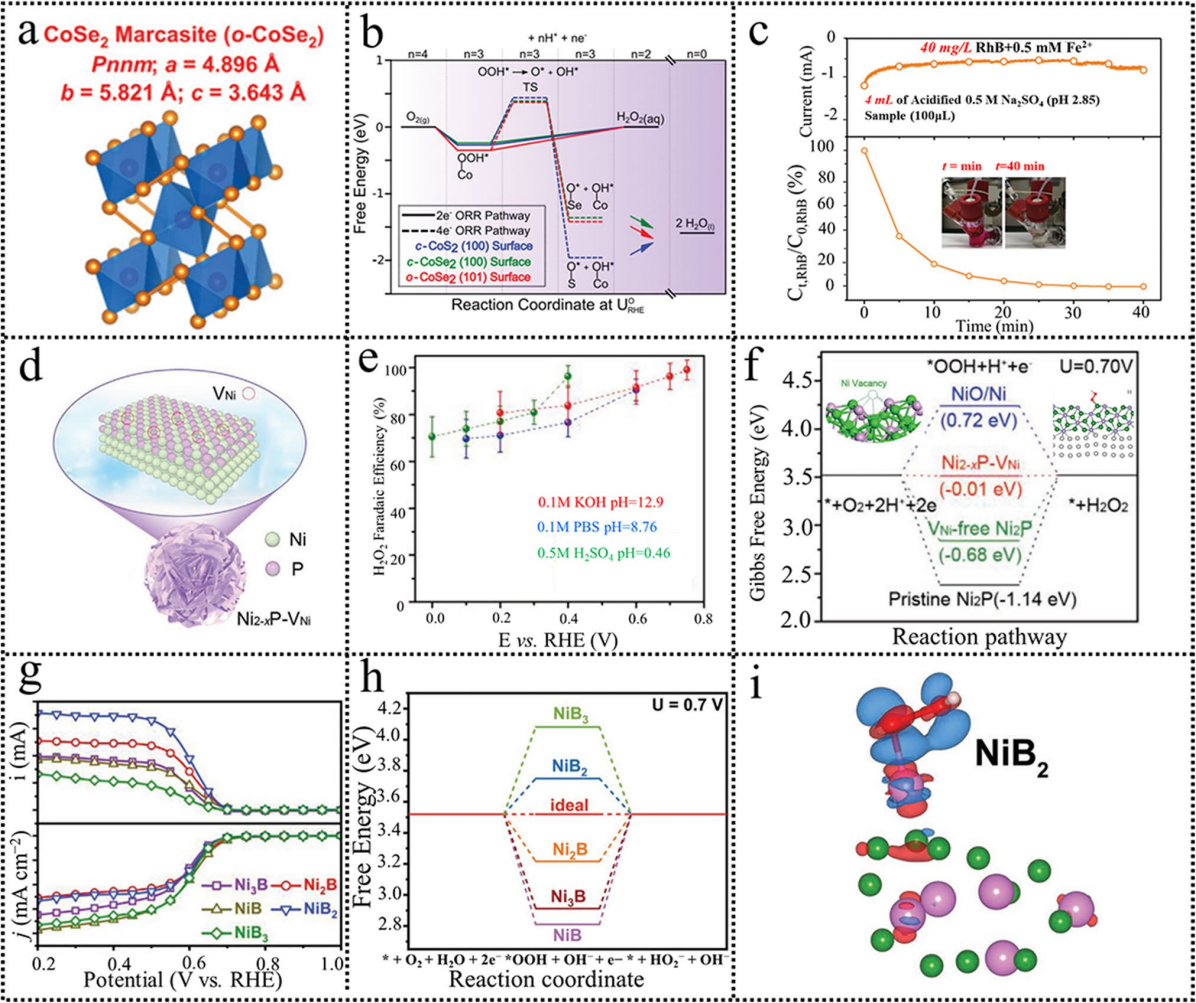
**a** Crystal structures, space groups, and lattice constants of *o*-CoSe_2_. **b** Calculated free energy diagrams of the 2e^−^ and 4e^−^ ORR pathways on the *c*-CoS_2_ (100), *c*-CoSe_2_ (100), and *o*-CoSe_2_. **c** Chronoamperometry curves of *o*-CoSe_2_/CFP at 0.5 V (*vs*. RHE). Reproduced with permission [99]. Copyright 2020, Royal Society of Chemistry. **d** Schematic illustration of Ni_2−*x*_P-V_Ni_. **e** The Faradaic efficiency of Ni_2−x_P-V_Ni_ in various electrolytes. **f** Free energy diagram of 2e^−^ ORR on all models at 0.70 V. Reproduced with permission [100]. Copyright 2022, Wiley. **g** LSV curves of the five Ni–B samples. **h** Free energy diagram for nickel boride clusters. **i** Charge density distribution of NiB_2._ Reproduced with permission [87]. Copyright 2022, Wiley. (Color figure online)

Motivated by this, they further investigated the effect of homotopy Se embeds in Co lattice on the electrosynthesis of H_2_O_2_ (Fig. 9a) [99]. It was found that the embedded Se atom had a larger atomic radius than the S atom, which can efficiently enable the separation of the neighboring Co active sites. The computational results were given to elucidate the ORR activity and H_2_O_2_ selectivity. The first step of *OOH formation was 0.27, 0.24, and 0.35 eV, corresponding to the *c*-CoS_2_ (100), *c*-CoSe_2_ (100), and *o*-CoSe_2_ (101) surfaces, respectively (Fig. 9b). This result suggested all these three samples were intrinsically active toward the 2e^−^ ORR. The 2e^−^ ORR selectivity was determined by the capability of O−O bond cleavage in the intermediate *OOH. The *o*-CoSe_2_ (101) showed a higher OOH* dissociation barrier (0.72 eV) than the *c*-CoS_2_ (100) and the *c*-CoSe_2_ given as the calculated results. Overall, the DFT results suggest that all CoSe_2_ are intrinsically electroactive and selective toward H_2_O_2_ electrosynthesis. Consequently, the *o*-CoSe_2_ exhibited much more effectiveness than CoS_2_ for the amply electrosynthesis of H_2_O_2_ and the accumulated H_2_O_2_ can effectively remove the RhB (Fig. 9c) [24]. Besides Co compounds, Ni compounds [100] were also studied as highly efficient and selective catalysts for the electrosynthesis of H_2_O_2_. Ni vacancies (V_Ni_)-enriched Ni_2−*x*_P–V_Ni_ electrocatalyst was fabricated as the schematic (Fig. 9d). The as-fabricated electrocatalyst exhibited excellent 2e^−^ ORR performance with a FE over 95% in 0.1 M KOH (Fig. 9e). DFT theoretical calculations were performed to investigate the enhanced H_2_O_2_ production performance on the Ni_2−x_P-V_Ni_ electrocatalyst. The closest distance of two Ni atoms in the Ni_2−*x*_P–V_Ni_ (3.72 Å) is much larger than that of the Ni_2_P (≈2.61 Å), far from the distance of O–O bond in *OOH intermediate (≈1.52 Å). Therefore, the *OOH adsorption on Ni_2−x_P–V_Ni_ prefers end-on bonding, promoting H_2_O_2_ electrochemical production. The calculated free energy on the Ni_2_P and Ni_2−*x*_P–V_Ni_ is − 1.14 and − 0.68 eV, respectively (Fig. 9f). The Ni_2−x_P-V_Ni_ delivered the lowest energy deviation (− 0.01 eV), demonstrating the best 2e^−^ ORR thermodynamics. Moreover, the Ni_2−x_P-V_Ni_ exhibited optimal binding energy of *OOH (ΔG_*OOH_ = 4.21 eV) with end-on adsorption, approaching the volcano apex (4.22 eV). The theoretical calculations and experimental results demonstrated that the Ni vacancies facilitated the electrochemical production of H_2_O_2_. Additionally, the practical H_2_O_2_ production of Ni_2−x_P-V_Ni_ in 0.1 M KOH, 0.1 M PBS, and 0.5 M H_2_SO_4_ was evaluated by flow cell technique. The FE in 0.1 M KOH was 80.7%, higher than that in 0.1 M PBS (71.1%) and 0.5 M H_2_SO_4_ (77.1%). These results proved that neutral electrolyte was not conducive to the oxygen selectively electrochemically reduction to H_2_O_2_, which is in consistent with the OHP and IHP mechanisms. Following this, various types of metal compounds such as metal oxides and metal borides are also fabricated for H_2_O_2_ electrosynthesis [101–104].

Currently, amorphous transition metal-based materials are found to own more intrinsic active sites and higher electrocatalytic activity compared to their crystalline counterparts due to their short-range order, unique electronic structure, and more abundant defects [85, 87]. Although there are very limited studies on amorphous metal compounds as catalysts for 2e^−^ ORR, the traces demonstrate that the amorphous metal compounds materials can be competitive catalysts. Kang et al. [87] found that amorphous nickel borides (Ni_3_B, Ni_2_B, NiB, NiB_2_, and NiB_3_) showed better H_2_O_2_ electrosynthesis performance than the previously reported crystalline nickel borides (Fig. 9g). In situ Raman revealed the main active sites are Ni. Especially for the amorphous NiB_2_, it delivered excellent electroactivity and H_2_O_2_ selectivity close to 100%, superior to the state-of-the-art transition metal compounds. Moreover, the amorphous NiB_2_ exhibited superior stability decay after undergoing a prolonged time test. DFT calculation demonstrated that the ∆G_OOH*_ (3.75 eV) value was approaching to the optimal value of 3.52 eV, indicating an optimal adsorption/desorption ability for *OOH (Fig. 9h, i). The optimal adsorption ability of *OOH can protect the O–O bond, in favor of H_2_O_2_ production. Furthermore, the higher ratio of Ni to B (such as for Ni_3_B, Ni_2_B, and NiB) induced a side-on model adsorption for *OOH presented, leading to a 4e^−^ ORR. However, when the ratio of B to Ni was increased, the B atoms could effectively isolate the neighboring Ni atoms, which promoted an end-on adsorption model of *OOH, in favor of the preservation of O–O bond and H_2_O_2_ production. Following this, Zhang and Hong et al. [85, 103] noted that the amorphous metal oxides (a-NiO) also show a balanced electrocatalytic activity and selectivity toward H_2_O_2_ production. The amorphous NiO nanosheets (a-NiO NSs) show much higher H_2_O_2_ selectivity than that of crystalline NiO nanosheets (c-NiO NSs), although the electrocatalytic activity is slightly lower. The ORR activity of a-NiO NSs can be increased by tuning short-range order and manufacturing more defects. The amorphous materials are competitive 2e^−^ ORR catalysts superior to their crystalline counterpart. It can be concluded that tuning short-range order in the amorphous materials can enhance more numbers of intrinsic active sites and induce unique electronic structure, thus optimizing the ORR activity. Moreover, apparent unsaturated coordination atoms in amorphous materials promote more defects, furthering increasing the ORR activity. Amorphous materials with high ORR activity and 2e^−^ ORR selectivity can be achieved for H_2_O_2_ electrosynthesis.

#### Atomically Dispersed Transition Metal Catalysts

The high cost of metal complexes and unsatisfactory electrical conductivity of metal compounds largely prohibited their practical applications [105, 106]. Nevertheless, some studies also reported the significant catalytic activity and durability improvement of metal complexes through heat treatment, tracing back to 1964 [107]. The coordination metal-heteroatom (e.g., Co-N_4_, Fe-N_4_) structure can be maintained, and remarkable ORR performance is achieved [106, 108–111]. Inspired by this, researchers are dedicated to transitional metal-nitrogen-doped carbon (M–N–C, M = Fe, Co, Mn, Cu, Ni) materials, as a promising alternative to the conventional noble metal-based catalysts (e.g., Pd, Au, or Pt) [112, 113]. M–N–C materials exhibit high activity comparable to the noble metal-based catalysts with much lower cost [74, 114–117]. Normally, their ORR activity and selectivity are affected by the central metal atoms, coordination heteroatoms, and other environmental atoms. It is universally believed that central metal atoms are active sites. Therefore, the selection of central metal atoms plays a crucial role in determining the ORR activity and selectivity.

**Fig. 10:**
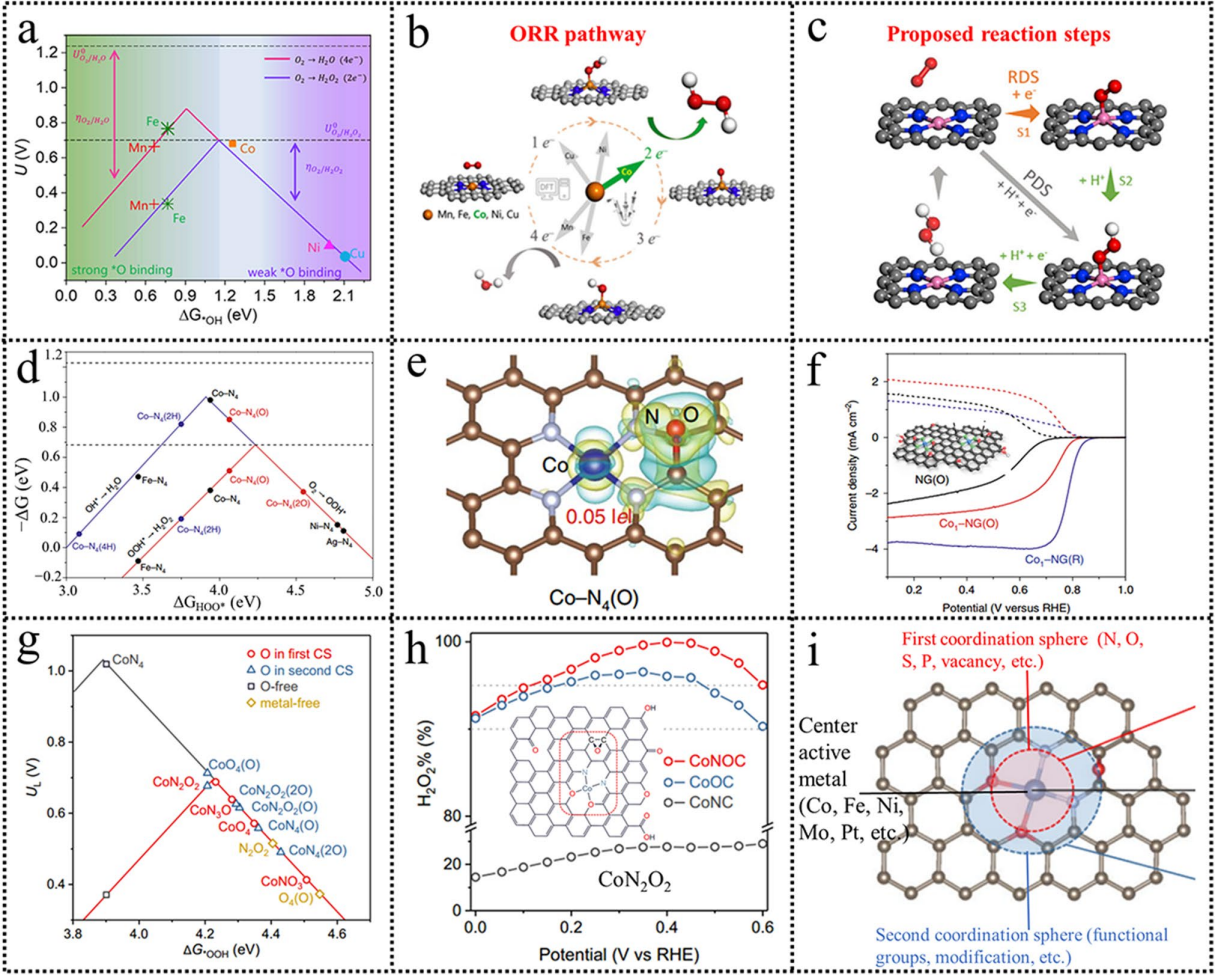
**a** Activity-volcano curves of ORR on M–N–C. **b** Schematic of the ORR pathway on M–N–C. **c** Proposed reaction steps of H_2_O_2_ synthesis over Co-NC. Reproduced with permission [119]. Copyright 2020, Elsevier. **d** Calculated catalytic activity volcanoes for the production of H_2_O (blue) and H_2_O_2_ (red) via the ORR (bottom panel). **e** Differential charge densities of Co–N_4_(O). **f** Comparison of ORR performance for NG(O), Co_1_–NG(O) and Co_1_–NG(R) catalysts. Reproduced with permission [120]. Copyright 2020, Springer Nature. **g** Computed activity volcano plots of ORR via the 2e^−^ (red color) or 4e^−^ (black) pathway for SACs with varied configurations. **h** Calculated H_2_O_2_ selectivity (H_2_O_2_%). **i** Schematic of SACs, highlighting the first and second coordination spheres and center active metal. Reproduced with permission [114]. Copyright 2021, American Chemical Society. (Color figure online)

In M–N-C materials, 3d transition metals with different d orbital electron numbers render them different d band centers, which can greatly affect the adsorption and desorption of reaction intermediate during the electrocatalysis process. Therefore, the selection of the central metal atom is extremely crucial to tuning the adsorption energy of the reaction intermediate. Strasser’s group [118] studied a series of 3d transition metals (Mn, Fe, Co, Ni, and Cu) over M–N–C materials through DFT calculation. Calculated results revealed that the Co–N–C catalyst had an optimal *OOH adsorption energy located on the top of the 2e^−^ ORR volcano. Fe–N–C and Mn–N–C catalysts possessed strong adsorption ability for *OOH, which might result in a predominant 4e^−^ ORR pathway. While for Cu–N–C and Ni–N–C catalysts, the binding of *OOH was too weak, which could not perform the ORR process efficiently. To verify the reliability of the calculation and unveil the trends in electrochemical H_2_O_2_ synthesis, a series of M–N–C catalysts were constructed by ball milling of metal precursors and successive high-temperature pyrolysis. It revealed that Co–N–C material exhibited the best 2e^−^ ORR performance, consistent with the DFT calculation results. The real H_2_O_2_ productivity over the Co–N–C catalyst was also measured in a microflow cell, reaching 4 mol peroxide g_catalyst_^−1^ h^−1^ at a current density of 50 mA cm^−2^. Subsequently, Liu’s group [119] also demonstrated that the Co–N–C catalyst was an optimal 2e^−^ ORR catalyst with moderate ORR activity among a series of M–N–C (Fe, Co, Mn, Ni, Cu) catalysts combined DFT calculation and experiment (Fig. 10a, b). In their research, the ORR pathway was revealed by adopting kinetic analysis. The results showed that the kinetic current of ORR and reaction orders of O_2_ were related to O_2_ partial pressure, pH, and the applied overpotential. The O_2_ partial pressures experiment suggested that the overpotential increased with the reaction order of O_2_ increasing. The pH effect experiment results showed that the reaction order of H^+^ was approaching zero, suggesting that H^+^ was not involved in the rate-determining step. The in situ techniques confirmed that the active sites were Co centers. The ORR step is illustrated in Fig. 10c, suggesting that the electron transfer step of adsorbed O_2_ (* + O_2_ → *O_2_^−^) was the rate-limiting step, while the protonation process was fast (* + O_2_ + H^+^  + e^−^  → *OOH) included in the DFT calculations. The electron transfer step is accelerated with the overpotential increasing. It is reasonable to assume that the overall reaction rate is more limited by the O_2_ adsorption process.

Environmental atoms are not directly connected to the central atoms. But the introduction of some functional groups into the local environment of metal-heteroatom-carbon is another effective way to indirectly change the electronic structure of active center atoms. For example, Hyeon et al. [120] calculated the ORR catalytic activity for the production of H_2_O and H_2_O_2_ of M−N_4_ (Fig. 10d). None of the M−N_4_ catalysts are proper for H_2_O_2_ production. It is significant to slightly modify M−N_4_ for H_2_O_2_ production while maintaining the high catalytic activity. Electron-rich species, such as O, adsorbed near the Co-N_4_ moiety (CoN_4_ (O)), could endow a positive charge state of the Co atom (Fig. 10e). While the electron-poor species, such as H^+^, adsorbed near the Co-N_4_ moiety (CoN_4_(H)) would enable it more negative. Consequently, electron-rich O species adsorbed near the Co-N_4_ moiety (Co-N_4_(O)) would increase the free energy of *OOH. ∆G_OOH*_ was increased to 4.1 eV after O* was adsorbed, approaching the optimal value for the H_2_O_2_ production (4.2 eV), which was favorable for the 2e^−^ ORR pathway. Electrochemical results revealed that the as-synthesized Co_1_-NG(O) catalyst exhibited high activity and 2e^−^ ORR selectivity (Fig. 10f). The pH effect was also studied, as shown in Table 1. The activity tendency of Co_1_-NG(O) was 0.1 M KOH > 0.1 M HClO_4_ > 0.1 M PBS. While the H_2_O_2_ selectivity tendency was different: 0.1 M KOH > 0.1 M PBS > 0.1 M HClO_4_, which could be due to the different ORR mechanism. Combining spectroscopic results and computational modeling, Lu et al. [121] demonstrated that the presence of epoxy groups near the Co-N_4_ centers was key to enhancing H_2_O_2_ productivity. In the electrochemical measurement process, they found that the selectivity of the CoN@CNTs catalyst toward H_2_O_2_ production falls dramatically after long-term exposure to air. O 1 s spectra showed that the epoxy groups had a drastic decrease, while the ketonic content in the aged CoN@CNTs sample increased significantly compared with the fresh CoN@CNTs. It was speculated that the epoxy groups would be critical to improving H_2_O_2_ productivity. Additionally, DFT calculation results also verified that the introduction of epoxy oxygen bonded to the Co-N_4_ configuration resulted in a weakened *OOH binding on the Co atoms. The binding energy of *OOH of Co-N_4_(2O) and Co-N_4_(3O) was located at the peak of the volcano for the 2e^−^ ORR. Batch experiments and DFT calculation results demonstrated that catalysts with Co-N_4_ configuration bonded to nearby epoxy groups showed superior activity and selectivity for H_2_O_2_ electrosynthesis. The performance is comparable to the state-of-the-art noble metal-based catalysts in acidic conditions. This work gives us an understanding of the basic relationship between the coordination environment of atomic sites and the H_2_O_2_ electrocatalytic properties for catalyst design.

An interesting discovery was recently reported in Qiao’s group [114]; they demonstrated that the neighboring C adjacent to the O could be the actual catalytic active sites in Co single-atom catalysts for the electrochemical H_2_O_2_ synthesis. Specifically, they adopted DFT calculations for the first time and demonstrated that the ORR activity and selectivity were mainly determined by modifying the first (N or/and O coordination) and second (C–O–C groups) coordination spheres. For CoN_4_, the optimized *OOH adsorption site is the center Co atom, while for CoN_2_O_2_ and CoO_4_, it is the C atom adjacent to the coordinated O atom. The *OOH binding on CoN_4_ was too strong (3.9 eV), very close to the 4e^−^ volcano peak. The *OOH binding was dramatically decreased after replacing coordination atoms N with O. The ΔG_*OOH_ values for CoN_2_O_2_ and CoO_4_(O) were 4.23 and 4.21 eV, respectively, approaching the peak of the 2e^−^ volcano (4.2 eV) (Fig. 10g). This, therefore, improves the 2e^−^ ORR to H_2_O_2_ generation due to the weaker *OOH adsorption. Three kinds of catalyst Co-N_4_-C configuration (derived from the metal–organic framework) and CoNOC/CoOC (Co^2+^ ions anchored on the oxidized CB followed by annealing under Ar with/without NH_3_) were precisely synthesized to verify the DFT calculations. Electrochemical results revealed that the CoNOC catalyst displayed the H_2_O_2_ selectivity nearly 100% over a wide range of potential with high activity, while CoNC exhibited a typical 4e^−^ ORR process (Fig. 10h). Combined thiocyanide (SCN^−^) poisoning experiment and in situ techniques confirmed that the actual active site of CoNOC was the C atom adjacent to the coordinated O atom. Based on this discovery, the authors concluded that the design principles for metal–heteroatom–carbon materials for H_2_O_2_ electrosynthesis are to regulate the binding strength of *OOH on different atoms by modifying the atomic configuration. The proposed molecular-level structure is given in Fig. 10i. Considering this, the active sites are adjustable via regulating the coordination atoms/environment.

**Fig. 11:**
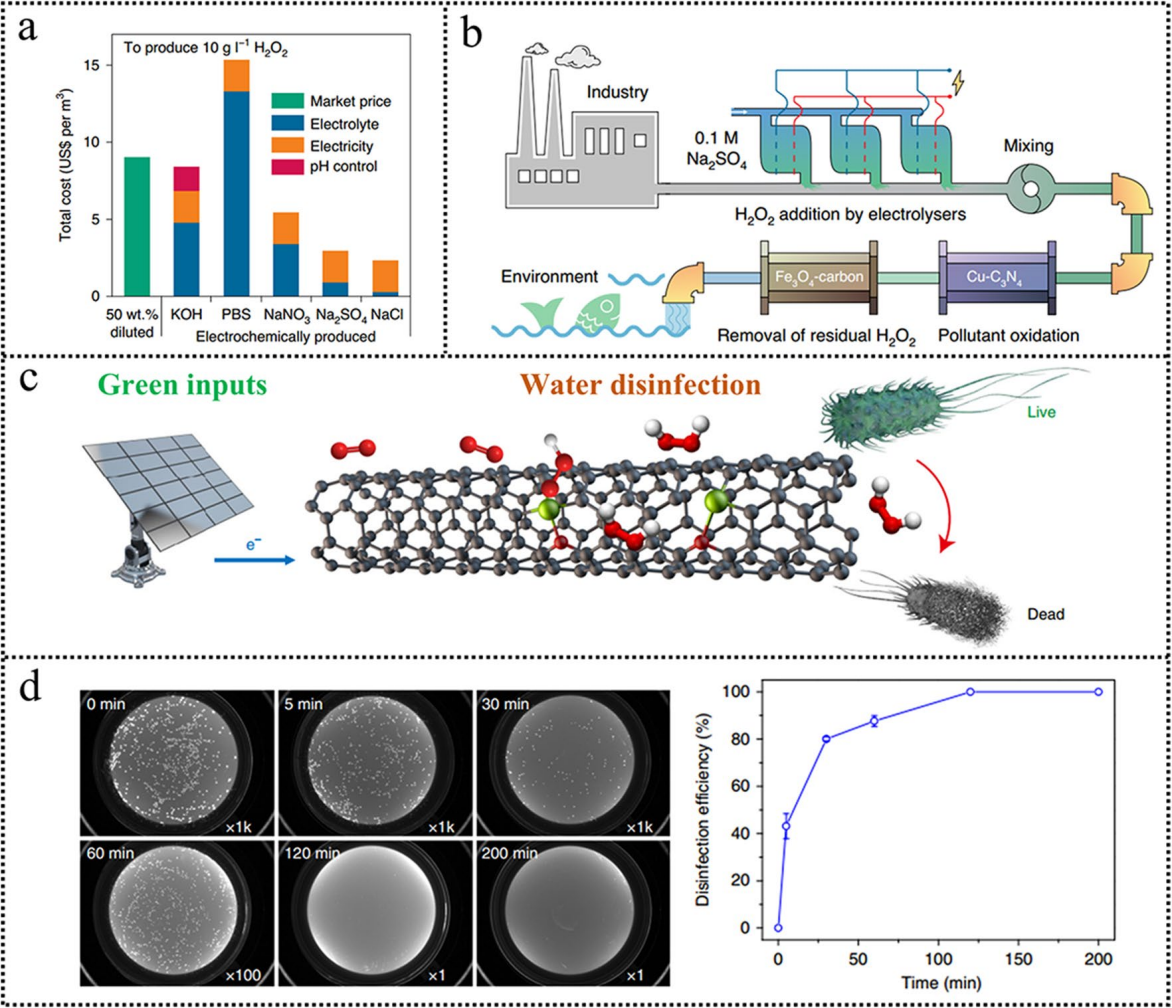
**a** Cost estimate for producing 10 g L^−1^ (1 wt%) H_2_O_2_ solution using different electrolytes (0.1 M). **b** Schematic drawing of the wastewater treatment system. Reproduced with permission [111]. Copyright 2020, Springer Nature. **c** Schematic of electrochemical synthesis of H_2_O_2_ for water disinfection, with green inputs such as sunlight, air, and water. **d** Water disinfection efficiency. Reproduced with permission [21]. Copyright 2019, Springer Nature. (Color figure online)

A wide range of practical applications could be realized with these low-cost transition metal-based catalysts for highly efficient H_2_O_2_ generation, involving organic contaminants degradation and disinfection. Organic wastewater poses considerable risks to the health of both humans and ecosystems. Cui et al. [117] demonstrated that Cu–N–C could produce H_2_O_2_ at a low cost. The total cost for producing 10 g L^−1^ H_2_O_2_ in 0.1 M Na_2_SO_4_ was only US$2.93 per m^3^, much lower than the traditional anthraquinone method (Fig. 11a).

Moreover, the produced H_2_O_2_ can treat the synthetic wastewater (containing 10 ppm triclosan, 17α-ethinyl oestradiol, and cefazolin sodium) to be below the detection limit. The zebrafish embryo teratogenicity analysis demonstrated that the effluent was safe for ecosystems (Fig. 11b). With the goal of global carbon neutrality, sustainable energy is the most ideal energy supply in the future. The electricity generated from wind and solar is an appropriate energy supply for the onsite production of H_2_O_2_ [21]. A wide range of practical applications could be realized with accessible inputs including sunlight for electricity, air for O_2_, and water as shown in Fig. 11c. The potential to deliver a 20 mA cm^−2^ constant current for H_2_O_2_ generation remained unchanged over the whole electrolysis course. The in situ producing H_2_O_2_ combined with the green electricity demonstrates a rapid disinfection efficiency delivering 43% bacteria inactivation in 5 min and more than 99.9999% in 120 min (Fig. 11d).

### Carbon-Based Catalysts

In the past decades, carbon-based materials have been widely studied in electrocatalysis due to their low cost, high surface area, and high conductivity [122–125]. It has been well accepted that the electrochemical performances of metal-free carbon-based materials are strongly determined by structures. The most effective and facile strategy is to endow the carbon materials with porous structures since a large surface area and high pore volume are beneficial for mass transfer and active sites exposed. Generally, the pristine carbon materials show poor ORR performance because of electroneutral carbon atoms. It is important to activate the inert carbon surfaces by creating defective carbon sites and doping heteroatoms.

#### Porous Carbon-Based Materials

**Fig. 12:**
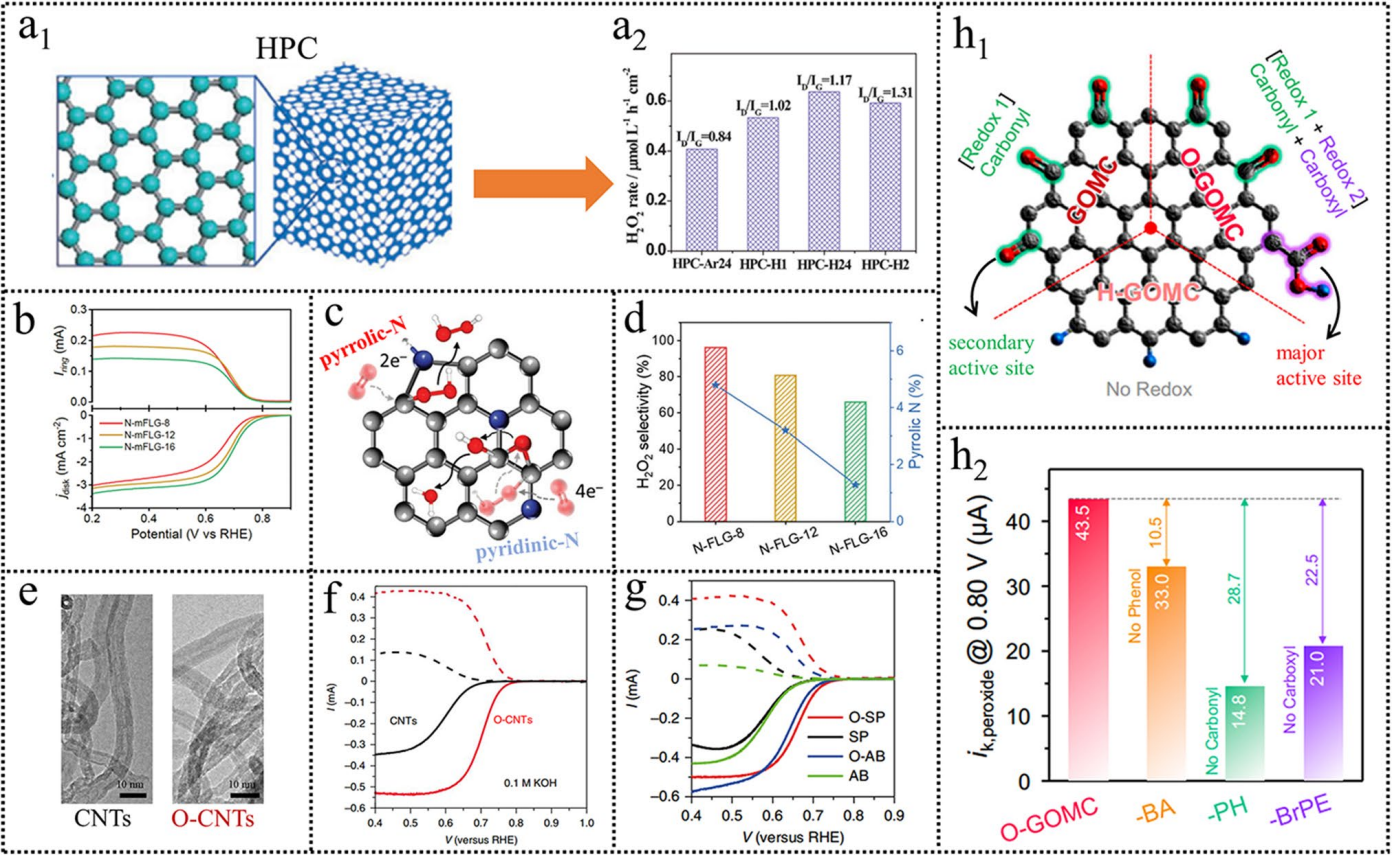
**a** Schematic illustration of HPC materials and their H_2_O_2_ production rates. Reproduced with permission [131]. Copyright 2015, Wiley. **b** ORR performance of N-FLG-8, N-FLG-12, and N-FLG-16. **c** Schematic diagram of two-electron and four-electron ORR pathways on N-FLG with different nitrogen configurations. **d** Relationship between H_2_O_2_ selectivity and atomic content of pyrrolic-N. Reproduced with permission [141]. Copyright 2020, Wiley. **e** TEM images of CNTs and O-CNTs. **f** ORR performance comparison of CNTs and O-CNTs. **g** ORR performance comparison of SP, O-SP, AB, and O-AB. Reproduced with permission [146]. Copyright, 2018, Springer Nature. **h** Illustration of active species in H-GOMC, GOMC, and O-GOMC. Reproduced with permission [132]. Copyright 2021, Elsevier. (Color figure online)

The most widely utilized structure regulation strategy is well-developed pore structure, pores volume, large surface area, and so on [126–129]. The structural properties play a substantial role during the electrocatalytic reaction process because they are closely linked to mass transport and the utilization of active sites [130]. For instance, a hierarchically porous carbon (HPC) derived from the metal–organic frameworks (MOFs) was presented for H_2_O_2_ electrosynthesis (Fig. 12a) [131]. The obtained HPC exhibited high catalytic activity and selectivity for electrochemical reduction of O_2_ to H_2_O_2_ over a wide pH range (1−7). The HPC catalyst with abundant micro-, meso-, or even macropores endowed it with plentiful exposed catalytic sites and shortened diffusion paths. Micropores could expose more active sites and provide additional active sites for ORR. The meso- and macropores allowed for fast transport of H_2_O_2_ from the catalyst surface to the bulk solution and reduced residence time, thus avoiding H_2_O_2_ further reduction to H_2_O. Especially for the carbon materials with a layered structure, most of the catalytic sites are buried in carbon layers in which interlayer spacing is too small to expose the catalytic sites. Using a template (silica, MgCl_2_, mesoporous aluminosilicate, and son) can create micropores or mesopores after template removal and thus expose more active sites [132]. Joo et al. [133] demonstrated that graphitic ordered mesoporous carbon (GOMC) nanocatalyst could expose abundant edge sites via mesoporous silica template introduction, rendering it with 28 times higher mass activity than that of a basal plane-rich CNT. Lee et al. [134] also demonstrated that 3D crumpled graphene showed an increased active surface area and could expose most of the buried active sites within 2D carbon layers through the MgCl_2_ introduction.

#### Heteroatom Doping

Porosity has been demonstrated to affect the performance of pristine carbon catalysts. The incorporation of heteroatoms (such as N, B, and P) into the carbon framework also is shown to be an effective way to improve the H_2_O_2_ electrochemical production performance. Altering the electronic structure of carbon atoms could result in a pronounced enhancement of both activity and selectivity for H_2_O_2_ production [135–140]. For N-doped electron-rich carbon nanostructures, the carbon π electrons can be activated by conjugating with the lone-pair electrons from N dopants. Thus, O_2_ molecules get reduced on the positively charged C atoms neighboring N. Qiao’s group [141] developed nitrogen-rich few-layered graphene (N-FLG) with a tunable nitrogen configuration for electrochemical H_2_O_2_ generation (Fig. 12b). The experiment results showed that the high nitrogen doping content could effectively alter the electronic structure and facilitate the O_2_ adsorption. Combined spectroscopic results and electrochemical performance suggested that the OOH* intermediates could be substantially preserved with the presence of a high amount of pyrrolic-N, leading to a 2e^−^ ORR pathway on the adjacent carbon atoms (Fig. 12c), while the 4e^−^ ORR pathway was supposed to preferentially occur on the carbon atoms adjacent to the pyridinic-N. Moreover, a positive correlation between the content of pyrrolic-N and the H_2_O_2_ selectivity was experimentally observed (Fig. 12d). Recently, Wang et al. [142] reported a series of nonmetal dopants, including B, N, P, and S, were anchored on a carbon support, and the resulting catalysts were screened for H_2_O_2_ electrosynthesis. The electrochemical results indicated that all doping samples showed enhanced performance than pure C. Among those candidates, the boron-doped carbon (B–C) catalyst presented the best performance with high activity (saving more than 210 mV overpotential) under industrial-relevant currents (up to 300 mA cm^−2^). Moreover, it maintained high H_2_O_2_ selectivity (85%–90%) compared with the state-of-the-art carbon catalyst. DFT calculations revealed that the boron dopant site is responsible for high activity and H_2_O_2_ selectivity due to low thermodynamic and kinetic barriers. The author further studied the pH effect on the H_2_O_2_ production. The B–C catalyst showed higher H_2_O_2_ selectivity up to 90% than that in 0.1 M Na_2_SO_4_ (80%) when tested by the RRDE technique, which is due to the lack of hydroxyl species. However, the ORR activity and the H_2_O_2_ selectivity show negligible difference when evaluated by the flow-cell device. It was noted that the solid-state electrolyte resulted in dramatically increased activity and H_2_O_2_ selectivity, originating from the decreased distance between the cathode and the anode. These results all illustrate that for electron-rich doping (such as N), the O_2_ molecules get reduced on the positively charged C atoms neighboring N, while for electron-deficient doping (such as B), O_2_ molecules are reduced on the positively charged B sites. Two main points to transform inert *sp*^2^ carbon into active metal-free ORR catalysts by heteroatomic doping can be summarized: (1) breaking the electroneutrality of *sp*^2^ carbon to create charged sites favorable for O_2_ adsorption despite whether the dopants are electron-rich (as N) or electron-deficient (as B) and (2) maximizing the effective O_2_ through activating carbon π electrons.

Except for single atom doping, co-doping/multi-doping could further optimize the carbon-based metal-free electrocatalysts [143]. For example, Chen et al. [139] presented the synergic mechanism of the doped N and F atoms in the process of electrocatalytic H_2_O_2_ electroproduction. Nitrogen- and fluoride-co doped carbon nanocages (NF-Cs) showed excellent electrocatalytic performance for H_2_O_2_ electroproduction with high FE both in alkaline solution (pH 13) (89.6%) and in acid solution (pH 0.35) (88%). The strong synergistic effect between the doped N and F atoms facilitated the H_2_O_2_ electroproduction. The doped N atoms promoted O_2_ molecule adsorption on the catalyst surface, while the F atoms facilitated the desorption of the *OOH intermediate, thus enhancing the catalytic activity and selectivity for H_2_O_2_ production. Moreover, different electronegative elements co-doping strategy was found to have a distinct influence on the catalytic activity and selectivity toward H_2_O_2_ production. Hu et al. [123] investigated B and N co-doped *sp*^2^ carbon materials. The experimental and theoretical results jointly indicated that when B and N were bonded together, doping hardly influences the electronic structure of carbon atoms and the CNTs material remained inert. The B and N co-doping could turn CNTs into excellent ORR electrocatalysts when the B and N atoms were separated. This phenomenon could be explained by the distinguish electronegativity of B and N atoms. The separation of B from N prevented the neutralization because N is the electron donor while B is the acceptor, so they were still capable of conjugating with the *sp*^2^ carbon, as in the single-doping. These results imply that the heteroatomic doping strategy plays a pivotal role in regulating this activity–selectivity dilemma. The essence of heteroatom doping and defects construction is to break the electroneutrality of *sp*^2^ carbon to create charged sites favorable for O_2_ adsorption.

#### Oxygen Functionalization

In addition to heteroatomic doping, oxygen functionalization strategies prove to be a facile but powerful method to promote electrochemical H_2_O_2_ production. The oxygen species can modulate the electronic structure of carbon, thereby controlling the binding energy of OOH* intermediate species for optimal electrochemical H_2_O_2_ production. There are many methods to introduce oxygen functional groups to carbon-based materials, such as acid surface oxidation, H_2_O_2_ oxidation, alkaline treatment, and oxygen plasma treatment [144–148].

Cui and co-workers [146] demonstrated a facile and general approach to carbon catalyst development via surface oxidation to enhance both the activity and for H_2_O_2_ selectivity (Fig. 12e). The H_2_O_2_ production performance of oxidized CNTs (OCNTs) was studied in 0.1 M KOH, 0.1 M PBS, and 0.1 M HClO_4_. The H_2_O_2_ selectivity was increased to 90% with a higher onset potential of 0.80 V in 0.1 M KOH (Fig. 12f). It was found that both the activity and selectivity of CNTs were positively correlated with the oxygen content of the catalysts. The ORR activity and H_2_O_2_ selectivity tendency were: 0.1 M KOH > 0.1 M PBS > 0.1 M HClO_4_ (Table 1). Introducing oxygen functional groups by surface oxidation was also effective for other carbon materials (Fig. 12g). Combined experiment results and DFT calculations, they assigned the carbon atoms adjacent to several oxygen functional groups (–COOH and C–O–C) as the active sites for 2e^−^ ORR. They also found that KOH and poly(ethylene oxide) (PEO) treatment could increase the oxygen functional group. It was noted that the activity and selectivity via KOH treatment were comparable with nitric acid surface oxidation. Kim et al. [149] reported a highly efficient electrocatalyst for H_2_O_2_ production through mild thermal reduction of graphene oxide. By using the in situ Raman technique, they identified the *sp*^2^-hybridized carbon near-ring ether defects along sheet edges as the most active sites. Most recently, Han et al. [150] prepared the quinone-enriched carbon catalyst as an excellent catalyst for H_2_O_2_ production, and the most active motif of quinone functional groups in the edge/basal plane was determined using DFT calculations. Oxygen functionalization processes usually create a variety of surface oxygen functional groups, whose type and relative distribution are generally uncontrollable. Therefore, the true active sites for 2e^−^ ORR are still elusive.

To understand the true active sites of oxygen-functionalized carbon materials, Liu et al. [123] described a chemical titration strategy to discriminate the H_2_O_2_ production activity for different oxygen functional groups. The oxygen-doped carbon nanosheets (OCNS700, OCNS800, OCNS900, and OCNS1000) were chosen as model catalysts. The OCNS900 exhibited excellent 2e^−^ ORR performances with a mass activity of 14.5 A g^−1^ at 0.75 V (*vs*. RHE) and remarkable H_2_O_2_ productivity in a flow cell with an H_2_O_2_ production rate of 770 mmol g^−1^ h^−1^. The electrochemical results combined with selective chemical titration experiments indicated that the C=O species were assigned as the most active sites for H_2_O_2_ electrosynthesis. Following this progress, Joo et al. [132] further constructed a systematic study to identify the catalytically active oxygen functional groups with controlled oxygen functionalities while fixing other structural properties. The electrochemical tests showed that the activity follows the same trend with the number of carboxyl groups (GOMC < O-GOMC-LT < O-GOMC), whereas their Tafel slopes and H_2_O_2_ selectivity were almost similar. The relationship between the surface functionality and 2e^−^ ORR activity was indicative of the carboxyl group at the edge sites of graphitic carbons as the major active site for the 2e^−^ ORR, and the carbonyl group as a secondary active site (Fig. 12h).

## Electrodes, Reaction Cells, and Their Architecture

Complementary to research on developing promising catalysts is the equally important pursuit of engineering the catalyst and other cell components into an efficient device. The ideal electrode structure should have sufficient active sites for oxygen adsorption, fast mass migration allowing rapid oxygen diffusion and H_2_O_2_ desorption, and electron transfer as well as good stability. The cell design plays a great role in scaling up electrochemical cells from the laboratory scale to the industrial scale.

**Fig. 13:**
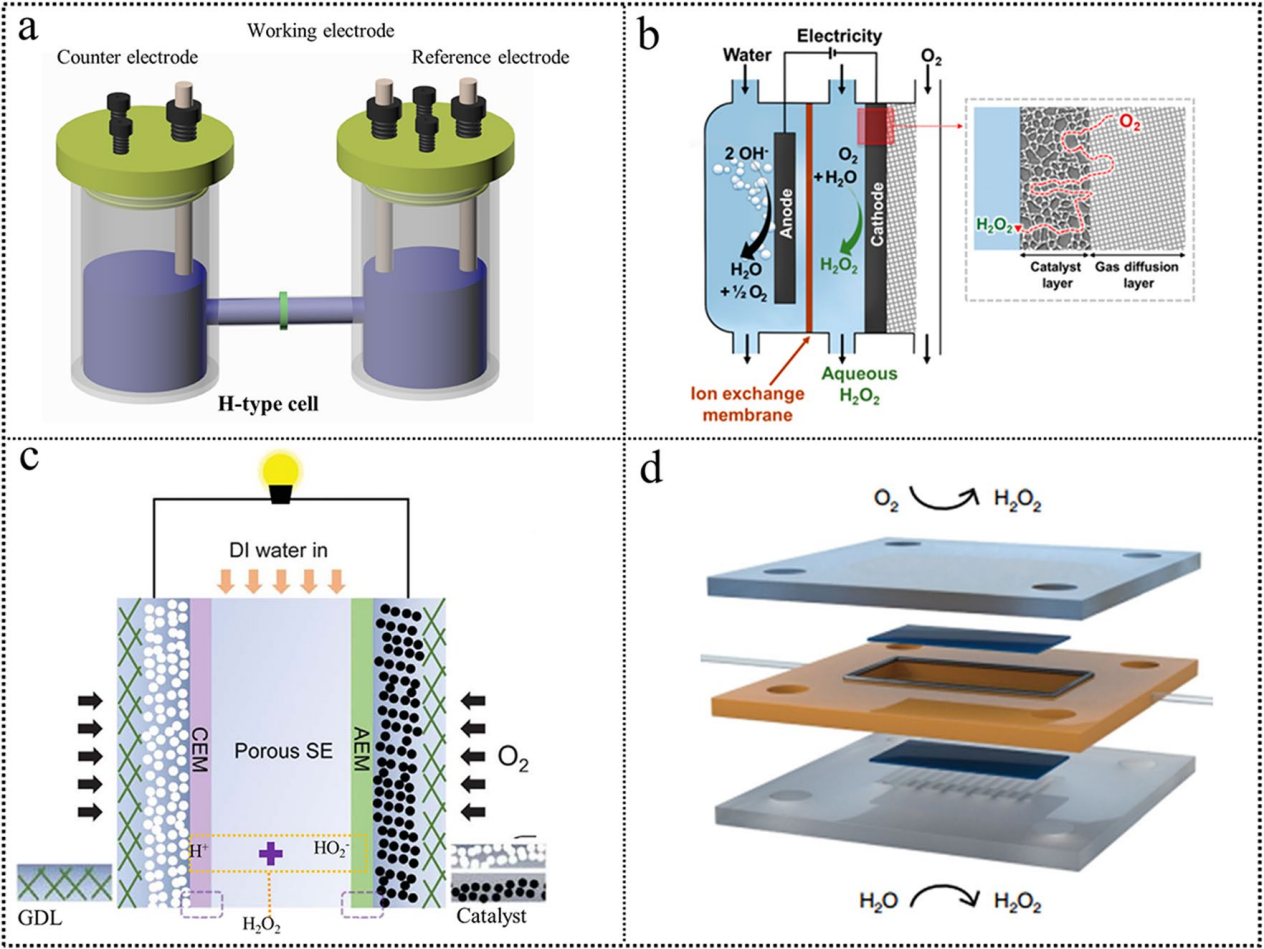
**a** H-cell device. **b** Schematic of a continuous flow cell with a catalyst deposited on a GDE. Reproduced with permission [27]. Copyright 2020, American Chemical Society. **c** Electrosynthesis of H_2_O_2_ using a solid electrolyte. Reproduced with permission [171]. Copyright 2019, American Association for the Advancement of Science. **d** Schematic design of developed 2e^−^-WOR//2e^−^-ORR H_2_O_2_ electrosynthetic cell. Reproduced with permission [174]. Copyright 2020, Springer Nature. (Color figure online)

The most common way to assess the H_2_O_2_ electrosynthesis performance is using a rotating ring-disk electrode [7]. However, the harsh test conditions make it impractical for a large-scale test. For practical applications, the H-cell setup was designed to evaluate the H_2_O_2_ electrosynthesis performance of large-area electrodes (Fig. 13a). Once the H-cell setup occurred, it received considerable attention for it was easy to operate and could generate bulk H_2_O_2_ [27, 151–154]. The electrode was submerged in the liquid electrolyte. Yamanaka et al. [155] studied the H_2_O_2_ production using an H-cell in a one-pot batch reactor. In this system, the reported H_2_O_2_ production reached a maximum value of 0.289 mmol cm^−2^ h^−1^ at the beginning. However, the H_2_O_2_ electrosynthesis performance quickly deteriorates, leading to low reaction rates, H_2_O_2_ concentrations, and FE. The H_2_O_2_ electrosynthesis performance showed a dramatic decay in a one-pot batch reactor compared to that tested in the H-cell with a membrane because the H_2_O_2_ in bulk can be decomposed on the anode surface. For the dual-chamber reactor, the cathode chamber and the anode chamber were separated with a proton-exchange membrane (PEM). However, the long distance between the cathode and the anode leads to the ion diffusion path increase accompanied by the increased solution resistance. Moreover, the low solubility of O_2_ in liquid electrolytes further limits the achievable H_2_O_2_ production rate. Although H-cells have been widely used for preliminary catalysts screening, they still cannot accurately evaluate how electrocatalysts and electrodes behave in industrial reactors because H-cells are not continuous and the accumulated H_2_O_2_ on the electrode surface leads to the further reduction of H_2_O_2_.

Initially, to solve the problem of the low solubility of oxygen in the electrolyte, the research has shifted to gas-diffusion electrodes (GDEs) that are composed of a hydrophobic layer [156–160]. These GDEs can act as a membrane between the oxygen gas and the liquid electrolyte. The GDEs are fabricated by depositing the catalyst on the gas diffusion layer and the three-phase interfaces (TPIs) accelerate the oxygen diffusion and maintain a constant O_2_ flow on the catalyst layer (Fig. 13b). [161, 162] The O_2_ can be electrochemically reduced to H_2_O_2_ as soon as it approaches the catalyst layer, promoting a high concentration of H_2_O_2_. This can effectively circumvent the problem of the low solubility of oxygen. The integrated electrodes with the advantages of fast electron transfer were constructed to reinforce the gas-diffusion channels against destruction by coating them with a thin gas-diffusion layer [163, 164]. Moreover, the established gas-diffusion layers can mitigate the H_2_O_2_ corrosion process towards the catalyst and the substrate, thus enhancing the stability and durability of catalysts. More importantly, recent studies show that PTFE treatment can slow down the H_2_O_2_ decomposition due to the decreased dielectric constant originating from the PTFE [108]. The substrates were required to supply gas flow channels to ensure the efficient contact between gas/catalyst and substrate/the catalyst [165–168]. The substrate originating from carbon showed superior compression strength, gas permeability, and corrosion resistance compared to the metal substrate.

For practical applications, the accumulated high concentrations of H_2_O_2_ near the electrode can be easily decomposed and accelerate electrode corrosion and catalyst degradation. Based on the practical experiences in water electrolysis and fuel cells for several years, the essential of promoting electrolyte flow for a long cycle is proposed. Dual-chamber reactor developed into a flow type for low-cost H_2_O_2_ electrosynthesis was reported by Chen et al. [169]. The catalyst loaded on commercial carbon support typically consisting of a GDL to enhance the O_2_ diffusion is used as a cathode. Studies demonstrate that O_2_ can effectively reach the catalyst surface. The continuous flow of reactants and products can prevent the accumulation of H_2_O_2_ near the electrode surface and accelerate the H_2_O_2_ inflow to the bulk electrolyte, promoting a higher current density accompanied by a higher reaction rate. Pérez et al. [170] reported that combined with a turbulence promoter, the efficiency in the flow cell is close to 100% with low energy consumption. The study revealed that the increase of the electrolyte inflow velocity could bring a progressive enhancement of the current density and H_2_O_2_ production. The flow cells make the H_2_O_2_ practical-scale production reliable.

Since the direct electrosynthesis of pure H_2_O_2_ solutions was up to 20 wt%, there has seen a revival of interest in H_2_O_2_ electrosynthesis. In Wang and colleagues’ work [171], a solid-electrolyte fuel cell was used to produce pure H_2_O_2_ solutions with H_2_ and O_2_ separately delivered to the anode and cathode, respectively. Protons transferred from the cation exchange membrane (CEM) and HO_2_^−^ ions transferred from the anion exchange membrane (AEM) were combined in the electrolyte chamber to generate H_2_O_2_ (Fig. 13c). In this case, the membrane between the chamber and the electrode avoids flooding in the case of direct contact of electrodes with water. The H_2_O_2_ concentrations can be varied by tuning the deionized water flow rate with no impurities introduced. Over 90% faradaic efficiency was achieved by using this device. Diverse “catholyte-free” flow reactors have been designed to obtain highly concentrated H_2_O_2_ via membrane electrode assembly, which combines the component of GDE, catalyst, and ion exchange polymer membrane into one unit. Based on the previously reported microfluidic cell, Kenis et al. [172, 173] pioneered a configuration without membranes that separate each side of the cell. Precise control over the cell assembly has been demonstrated to be effective in obtaining high current densities for H_2_O_2_ electrochemical production. Recently, Xia et al. [174] also manifested a high efficiency of the membrane-free flow cell for H_2_O_2_ electrocatalytic generation (Fig. 13d). The maximum FE jumped to 200% because the hydrophilic carbon fiber paper could be used as cathode and anode to simultaneously produce H_2_O_2_. The hydrophilic layer can decrease dielectric constant of the aqueous solution, thus increasing the H_2_O_2_ decomposition overpotential and slow down the H_2_O_2_ decomposition process [108]. When the cell current arrived at 50 mA cm^−2^, a cell voltage was only approximately 1.7 V, whereas 1.98 V cell voltage was required for conventional cells, sharply reducing the energy consumption. Thus, biomass conversion reaction may be chosen for electrochemical oxidation as an attractive alternative to traditional water oxidation, which can not only produce a valuable product but also reduce the cell voltage to make energy consumption decreased.

## Summary and Perspectives

**Fig. 14:**
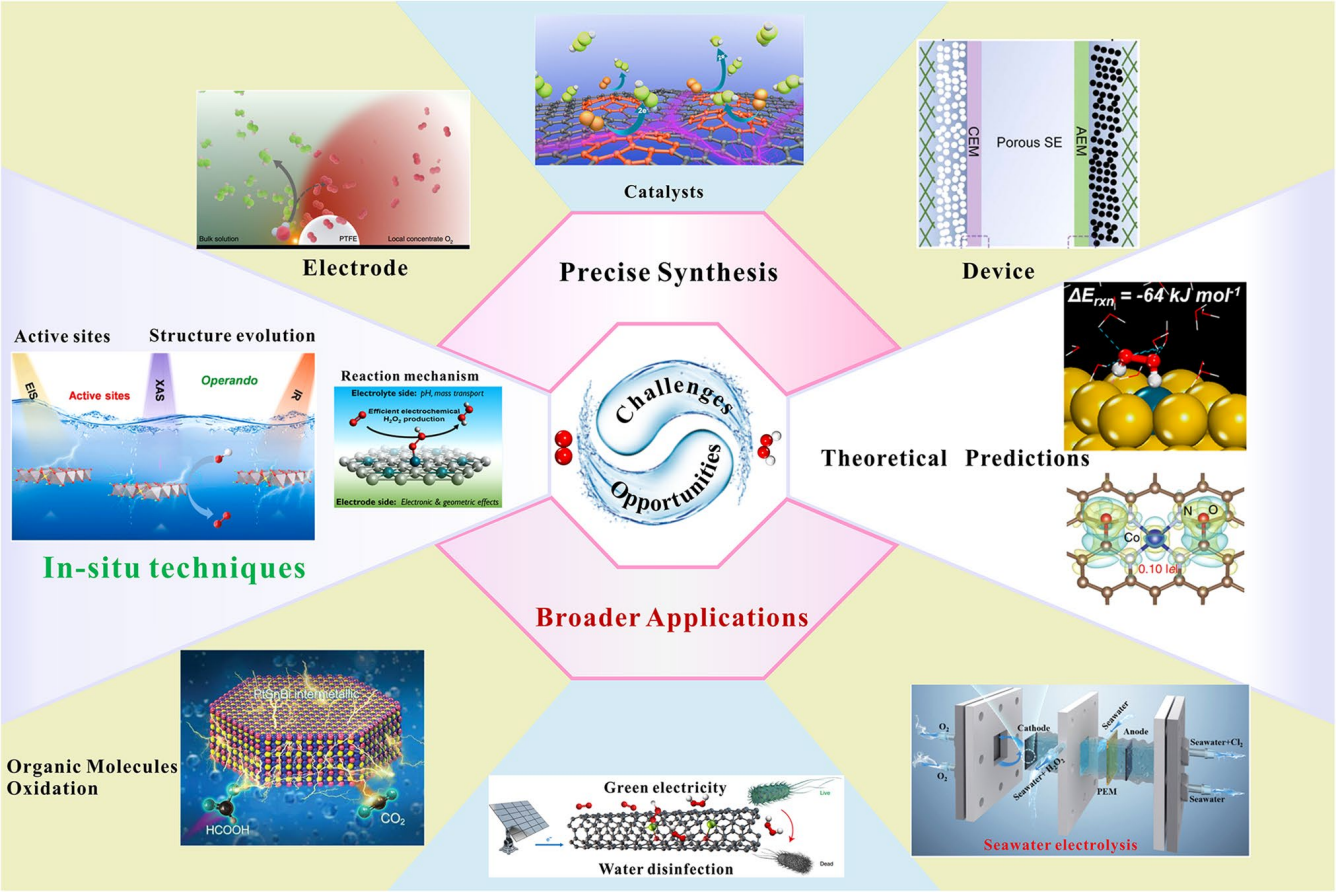
Illustration of the challenges and opportunities in the development of the H_2_O_2_ electrochemical production. (Color figure online)

In this review, significant advances in the development of electrocatalytic H_2_O_2_ production over various catalysts, electrodes, and other cell components are summarized and discussed in Table 1. SACs with lower cost are the most efficient catalysts among the available catalysts for H_2_O_2_ electrosynthesis. Much effort has been focused on the design and engineering of the electrode and electrochemical reactors to realize the industrial production of hydrogen peroxide via electrosynthesis. Despite significant advances, it is still in its infancy to realize the scale-up of H_2_O_2_ electrochemical production. Therefore, challenges and opportunities are present for the industrial production of H_2_O_2_ (Fig. 14).

1. Excavating ORR catalysts with new composition and structure is still at the heart of the H_2_O_2_ electrochemical production. To date, noble-based catalysts have been thought to be the most efficient ORR catalysts for H_2_O_2_ production. However, their high cost and scarcity restrict their industrial application. Thus, given the cost issue, precise synthesis of trace precious metal-based catalysts or precious metal-free-based high-efficient ORR catalysts is urgently needed. Recently, cost-effective amorphous materials and single-atom catalysts with maximally utilized active sites are emerging. Especially for the single-atom catalysts, they are regarded as the next-generation ORR catalysts with high ORR activity to enable the H_2_O_2_ electrochemical production large-scale [72, 84]. In the future, single atoms catalyst with amorphous structure can be expected to be explored. Catalysts must be deposited on the substrate by in situ growing on the substrate, electrodepositing process, or spraying the catalyst on the substrate. As one component of the device, the basic role of the substrate is to load the catalyst and collect the produced current. More importantly, they are required to provide gas flow channels to maximize the electrochemical utilization of gases. Metal substrates, such as SS316L, are easy to be corroded and the corrosion production increases the contact resistance. Therefore, more efforts should be devoted to developing good catalytic and corrosion-resistant electrodes and studying their effect on the industrial production of H_2_O_2_.
2. The currently developed in situ techniques can provide new insight into identifying the active sites and structure evolution, which is of critical significance for designing efficient 2e^−^ ORR catalysts. For example, to gain in-depth into the adsorbed oxygen intermediates on the catalysts during the ORR process, operando ATR-IR measurements could be used for characterizing OOH_ad_ and HOOH_ad_ to confirm the 2e^−^ ORR pathway [175]. In situ X-ray absorption near edge structure (XANES) and extended X-ray absorption fine structure (EXAFS) could be carried out to identify the change of coordination configuration and valence states near active sites [176]. In situ Raman spectroscopy could be used to probe the chemical bond stretching and bending vibrations.
3. With the development of computer technologies, thriving theoretical chemistry has been demonstrated as an innovative technology for identifying and developing new efficient catalysts and predicting the potential reaction mechanism combined with in situ technologies [12]. For example, Hyeon et al. predicted the reaction energetics of H_2_O_2_ electrochemical production on M–N_4_/graphenes (M = Co, Ni, Fe, Pt, Ag, and Ru) and Co–N_4_/graphene with 4H*/2H* and O*/2O* adsorbed near the cobalt atom where Co–N_4_(O) possessed the optimal OOH* adsorption energies [120]. It is attractive to utilize theoretical chemistry to seek out new catalysts.
4. Electrochemical production of H_2_O_2_ via 2e^−^ ORR using renewable energy is promising from the viewpoint of practical applications for water disinfection and wastewater treatment [177, 178]. The green electricity generated from solar and wind farms can realize a more efficient and cleaner onsite H_2_O_2_ production [20, 21]. Much of the literature about H_2_O_2_ production has focused on the cathode, while the anode is relatively neglected. Thus, we should combine the future study on H_2_O_2_ electrochemical production and seawater, oxidation of organic small molecules, which is more attractive than the conventional H_2_O_2_ electrosynthesis, integrating various applications [179–181].
